# Identification and assessment of variable single-copy orthologous (SCO) nuclear loci for low-level phylogenomics: a case study in the genus *Rosa* (Rosaceae)

**DOI:** 10.1186/s12862-019-1479-z

**Published:** 2019-07-24

**Authors:** Kevin Debray, Jordan Marie-Magdelaine, Tom Ruttink, Jérémy Clotault, Fabrice Foucher, Valéry Malécot

**Affiliations:** 1 IRHS, Agrocampus-Ouest, INRA, UNIV Angers, SFR 4207 QuaSaV, Beaucouzé, France; 2 ILVO, Flanders Research Institute for Agriculture, Fisheries and Food, Plant Sciences Unit, Melle, Belgium

**Keywords:** Species-level phylogenomics, Nuclear single-copy orthologs, Phylogenetic informativeness, Conflicting topologies

## Abstract

**Background:**

With an ever-growing number of published genomes, many low levels of the Tree of Life now contain several species with enough molecular data to perform shallow-scale phylogenomic studies. Moving away from using just a few universal phylogenetic markers, we can now target thousands of other loci to decipher taxa relationships. Making the best possible selection of informative sequences regarding the taxa studied has emerged as a new issue. Here, we developed a general procedure to mine genomic data, looking for orthologous single-copy loci capable of deciphering phylogenetic relationships below the generic rank. To develop our strategy, we chose the genus *Rosa*, a rapid-evolving lineage of the Rosaceae family in which several species genomes have recently been sequenced. We also compared our loci to conventional plastid markers, commonly used for phylogenetic inference in this genus.

**Results:**

We generated 1856 sequence tags in putative single-copy orthologous nuclear loci. Associated in silico primer pairs can potentially amplify fragments able to resolve a wide range of speciation events within the genus *Rosa*. Analysis of parsimony-informative site content showed the value of non-coding genomic regions to obtain variable sequences despite the fact that they may be more difficult to target in less related species. Dozens of nuclear loci outperform the conventional plastid phylogenetic markers in terms of phylogenetic informativeness, for both recent and ancient evolutionary divergences. However, conflicting phylogenetic signals were found between nuclear gene tree topologies and the species-tree topology, shedding light on the many patterns of hybridization and/or incomplete lineage sorting that occur in the genus *Rosa*.

**Conclusions:**

With recently published genome sequence data, we developed a set of single-copy orthologous nuclear loci to resolve species-level phylogenomics in the genus *Rosa*. This genome-wide scale dataset contains hundreds of highly variable loci which phylogenetic interest was assessed in terms of phylogenetic informativeness and topological conflict. Our target identification procedure can easily be reproduced to identify new highly informative loci for other taxonomic groups and ranks.

**Electronic supplementary material:**

The online version of this article (10.1186/s12862-019-1479-z) contains supplementary material, which is available to authorized users.

## Background

Next-Generation Sequencing (NGS) methods are now extensively used to address various scientific issues ranging from ecology to medicine, and become more affordable each year. Molecular phylogenetic studies greatly benefit from the high-throughput sequencing technologies that generate a wealth of information to decipher taxa relationships [1]. The 1000 plant (1KP) project [2] released large-scale gene sequencing data for over 1000 species, and thousands of other genome sequences are expected in the near future [3]. Relationships among angiosperms are relatively well-known, ranging from deep branches to the family rank [4, 5], with some exceptions [6]. However, it is often challenging to understand shallower relationships in particular angiosperm families, especially between species [7, 8]. Rapid diversifications are common to angiosperms, involving evolutionary processes such as polyploidization and hybridization [9, 10]. These two processes are likely to occur between closely-related species, generally inside genera [11]. While plant molecular phylogenetics has long been dominated by plastid sequence analysis [12, 13], identifying nuclear genes has now become an important issue in phylogenetic reconstruction, especially for hybrid and polyploid taxa [14]. Nuclear markers generally show higher rates of evolution than plastid sequences and may contain more informative nucleotide substitutions to distinguish closely-related taxa [15]. Whereas plastid genomes are mainly maternally inherited in angiosperms [16], nuclear markers contain sequence signatures of both parents, making them more useful to study hybridization and polyploidization events in taxa at the boundary between species and populations [15, 17]. Up to now, only few nuclear genes that are ubiquitously present in species across the Tree of Life have been commonly used for phylogenetics such as nuclear ribosomal internal transcribed spacers (nrITS) and glyceraldehyde 3-phosphate dehydrogenase (GAPDH). However, such sequences may present multiple issues for phylogenetic analyses. GAPDH is better suited to resolve relationships at the kingdom or class level [18, 19] than at the genus or species levels. nrITS exist in multiple copies that might not evolve at the same rate so that comparison between them may mislead phylogenetic analyses [20, 21]. With the ever-growing number of available whole genome sequences, several sets of new nuclear markers have been published to help unravel phylogenetic relationships at different plant taxonomic levels, ranging from the angiosperm clade [22–25] to particular families [26–28]. Specific attention has been given to single-copy genes (SCG) that go beyond the issues of conventional markers (ie plastid sequences or ubiquitous nuclear genes) and turn out to be good candidates for phylogenetic analysis [15, 29]. In addition to their biparental inheritance and their high content of informative characters, SCGs ease the identification of orthologs [15]. Orthologs are genes that derive from speciation events, as opposed to paralogs that derive from duplication events and should therefore be discarded from phylogenetic analyses. Consequently, sequences found in a wide range of taxa and that share a 1-to-1 homology with core SCGs may have resulted from speciation events and may therefore be considered as orthologous sequences. In angiosperm genomes, 8–35% of the genes are found as a single copy [24], providing the opportunity to find many orthologous sequences well suited to carrying out phylogenetic studies at various taxonomic levels.

Phylogenomics, i.e., the use of large arrays of genome sequences to infer phylogenetic relationships, has emerged over the last few years and is increasingly used in molecular studies of taxa relationships [30, 31]. With the tremendous increase in plant genome sequencing projects [32], it is now feasible to include thousands of sequences for phylogenetic analysis. Since a larger set of genomic sequences are included in the comparison, topological conflicts between individual gene trees and the species-tree arise [33–36]. These conflicts could be due to horizontal gene transfer, incomplete lineage sorting, and gene duplication and gene loss [37]. To circumvent these particular issues, a common method consists in concatenating the gene sequences, assuming that the true overall phylogenetic signal would arise and conceal the noise contained in individual genes [38, 39]. Several methods have been developed to assess this noise and to help in selecting the best marker set with the most informative characters captured with the lowest number of sequences. Most of these methods rely on distance metrics derived from tree topologies [40] and branch length comparisons [41, 42] or, alternatively, on likelihood ratio tests [43, 44] combined with various clustering methods [45–49]. Other methods use a conceptual index to assess the phylogenetic utility of sequences [50]. The main goal of marker selection is to find the optimal balance between character sampling and taxon sampling. Too few markers may lead to inaccurate estimations of phylogenetic relationships whereas too many markers increase the computational needs and the overall cost of the experiment, especially for phylogenomic studies involving a broad number of taxa.

Phylogenetic analysis of the genus *Rosa* is challenging because the genus comprises approximatively 150 species distributed in the Northern Hemisphere that are the result of a complex evolutionary history involving multiple hybridization and polyploidization events across the last 30 M years [51]. Currently, Rehder’s classification [52], slightly modified by Wissemann [53], is still used and divides the genus into four subgenera (*R*. subgen. *Rosa*, *R*. subgen. *Hulthemia* (Dumort.) Focke, *R*. subgen. *Platyrhodon* (Hurst) Rehder and *R*. subgen. *Hesperhodos* Cockerell). About 95% of the wild rose species belong to the subgenus *Rosa* which is further divided into ten sections (*R*. sect. *Pimpinellifoliae* (DC.) Ser., *R*. sect. *Gallicanae*, *R*. sect. *Caninae* (DC.) Ser., *R*. sect. *Carolinae* Crép., *R*. sect. *Rosa* [= *R.* sect. *Cinnamomeae* (DC.) Ser.], *R*. sect. *Synstylae* DC., *R*. sect. *Chinenses* Ser. [= *R*. sect. *Indicae* Thory], *R*. sect. *Banksianae* Lindl., *R*. sect. *Laevigatae* Thory and *R*. sect. *Bracteatae* Thory). In this paper, we adopt the designation of *Rosa cinnamomea* L. (syn. *Rosa majalis* Herrm.) as the type species of the genus, a proposal from Jarvis [54] and validated in 2005 at the Vienna International Botanical Congress. This implies that the section previously known as *Rosa* sect. *Cinnamomeae* (DC.) Ser. is renamed *R.* sect. *Rosa*. In addition, Wissemann [53] subdivided the *R*. sect. *Caninae* into six subsections (*R*. subsect. *Trachyphyllae* H. Christ, *R*. subsect. *Rubrifoliae* Crép., *R*. subsect. *Vestitae* H. Christ, *R*. subsect. *Rubiginae* H. Christ., *R*. subsect. *Tomentellae* H. Christ and *R*. subsect. *Caninae*). *R*. sect. *Caninae* is an evidence of rapid radiation in the genus *Rosa*. While this section accounts for approximatively 20% of the *Rosa* species, it appeared only ca. 6 MYa [51]. Thus far, the phylogenetic relationships among wild roses have been explored with nrITS [55–61], chloroplast regions [51, 59–65], and GAPDH [17, 51], as phylogenetic markers. The phylogenetic relationships derived from these conventional markers either focused on specific sections, or were poorly resolved, because many clades lacked support due to little sequence variation between the sampled species. Nevertheless, Fougère-Danezan et al. [51] distinguished three main clades (sect. *Synstylae* and allies, sect. *Pimpinellifoliae*, and sect. *Cinnamomeae* [i.e., sect. *Rosa*] and allies) and is currently the most completely resolved phylogeny of the genus *Rosa*. The recent publication of a high-quality reference genome sequence of *Rosa* ‘Old Blush’ [66, 67], a putative hybrid between *R. chinensis* and *R. odorata* va*r. gigantea* [68], provides an excellent resource to mine for nuclear sequences for high-resolution phylogenomic analysis of the genus *Rosa*. Moreover, multiple poor quality draft genomes of wild *Rosa* species have recently been released and can also be mined for shared loci with sequence variations between the different species (Table 1). We used these genomes here to present a general method to identify a set of single-copy nuclear orthologous loci that can be amplified from species across the genus. These sequences contain the sequence variations required to study species relationships through phylogenomics. The method was developed for the genus *Rosa*, and can be used at different taxonomic levels and groups.

**Table 1:** References used for Whole Genome Shotgun data

	Species	Ploidy of the genome sequence	Sample origin	BioProject/SRA code	Original publication
Ingroup	*Rosa* ‘Old Blush’	1x	IRHS, Beaucouzé, France	–	[67]
	*Rosa arvensis* Huds.	2x	Jardin expérimental de Colmar, Colmar, France	SRX3286288	[67]
	***Rosa chinensis*** **Jacq. var.** ***spontanea*** **(Rehd. & Wils.) T.T. Y & T. C. Ku**	2x	Roseraie du Val-de-Marne, L’ Hay-les-Roses, France	SRX4006790	[67]
	*Rosa* × *damascena* Mill.	4x	Bulgaria	PRJNA322107	–
	***Rosa gigantea*** **Collet ex Crép**	2x	Lyon botanical garden, Lyon, France	SRX3286284, SRX3286283	[66]
	***Rosa laevigata*** **Michx.**	2x	Roseraie du Val-de-Marne, L’ Hay-les-Roses, France	SRX4006792	[67]
	***Rosa majalis*** **Herrm.**	2x	ENS Lyon, Lyon, France	SRX3286287	[66]
	***Rosa minutifolia*** **var.** ***alba*** **Engelm.**	2x	Roseraie du Val-de-Marne, L’ Hay-les-Roses, France	SRX4006787	[67]
	***Rosa moschata*** **Herrm.**	2x	Roses Loubert rose garden, Les Rosiers-sur-Loire, France	SRX4006793	[67]
	*Rosa multiflora* Thunb. ex Murr.	2x	Keisei Rose Nurseries, Chiba, Japan	PRJDB4738	[69]
	***Rosa odorata*** **(Andr.) Sweet**	2x	Lyon botanical garden, Lyon, France	SRX3286293	[66]
	*Rosa palustris* Marsh.	2x	NA	ERS1829481	[70]
	***Rosa pendulina*** **L**	2x	Lyon botanical garden, Lyon, France	SRX3286278	[66]
	***Rosa persica*** **Michx. ex Jussieu**	2x	Roses Loubert nurseries, Les Rosiers-sur-Loire, France	SRX4006789	[67]
	***Rosa rugosa*** **Thunb.**	2x	Roseraie du Val-de-Marne, L’ Hay-les-Roses, France	SRX4006791	[67]
	*Rosa wichurana* Crép	2x	ILVO, Melle, Belgium	PRJNA504542	–
	***Rosa xanthina*** **va*r. xanthina*** **f.** ***spontanea*** **Rehd.**	2x	Roses Loubert rose garden, Les Rosiers-sur-Loire, France	SRX4006788	[67]
Outgroup	*Fragaria vesca* L.	1x	NCGR, Corvallis, OR, USA	PRJNA66853	[71]
	*Fragaria iinumae* Makino	2x	Kagawa University, Kagawa, Japan	PRJDB1478	[72]
	*Fragaria nipponica* Makino	2x	Kagawa University, Kagawa, Japan	PRJDB1479	[72]
	*Fragaria nubicola* Lindl. ex Lacaita	2x	NCGR, Corvallis, OR, USA	PRJDB1480	[72]
	*Geum urbanum* L*.*	2x	Punnets Town, UK	PRJEB23412	[73]
	*Potentilla micrantha* Ramond ex DC.	6x	Avala, Serbia	PRJEB18433	[74]
	*Rubus occidentalis* L.	2x	Rich Mountain, South Carolina, USA	–	[75]

Bold species indicate unassembled Whole Genome Shotgun data

*IRHS* Institut de Recherche en Horticulture et Semences, *ENS* École Normale Supérieure, *ILVO* Instituut voor Landbouw-, Visserij- en Voedingsonderzoek, *NCGR* National Clonal Germplasm Repository

## Results

### Identification of single-copy orthologs (SCO) in *Rosa* ‘Old Blush’ and *Fragaria vesca*

**Fig. 1:**
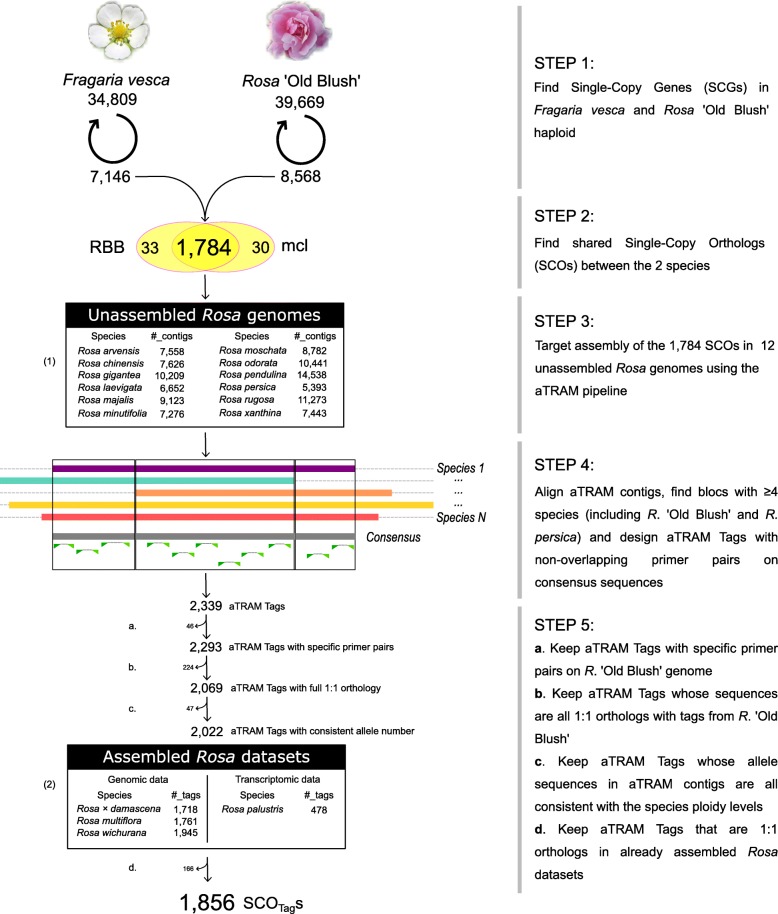
Data-mining workflow to identify single-copy orthologous tags (SCO_Tag_s) for phylogenomics. Single-copy genes (SCGs) from reference genomes are identified using a self-blast procedure (step 1). The two SCG sets are compared to each other to retrieve shared single-copy orthologs (SCOs) (step 2). SCOs are target-assembled from unassembled whole genome shotgun sequencing data using the aTRAM pipeline. Numbers presented in table (1) correspond to the total number of contigs that were assembled for each *Rosa* species with an unassembled genome (step 3). Contig sequences from each SCO are aligned using mafft and the resulting alignment is sliced in regions ≥300 bp covered by ≥4 taxa including *Rosa* ‘Old Blush’ and *Rosa persica*. For each region, pairs of primers are designed on the consensus sequence and the most variable non-overlapping SCO_Tag_s are retained (step 4). Additional filtering steps enables to discard SCO_Tag_s with unspecific primer pairs (step 5a), SCO_Tag_s that do not pass the RBB test of orthology (5b), SCO_Tag_s with inconsistent number of alleles regarding the genome ploidy level (5c) and to find SCO_Tag_s in whole genome shot gun assemblies of three additional *Rosa* species (step 5d) and seven outgroups. Numbers in table (2) correspond to the number of SCO_Tag_s that were retrieved for each of the four *Rosa* species with already assembled datasets. The procedure is described in detail in the Methods section. RBB: Reciprocal Best Blast; mcl: Markov CLuster algorithm

We compared annotated proteins from reference genomes of haploid *Rosa* ‘Old Blush’ [67] and *Fragaria vesca* [71] to identify single-copy orthologs (SCOs) using the all-against-all BLAST+ procedure. We found that *Rosa* ‘Old Blush’ (resp., *Fragaria vesca*) has 8568 single-copy genes (resp., 7146), which represents 21.6% (resp., 20.5%) of all predicted proteins for this genome (Step 1, Fig. 1).

**Fig. 2:**
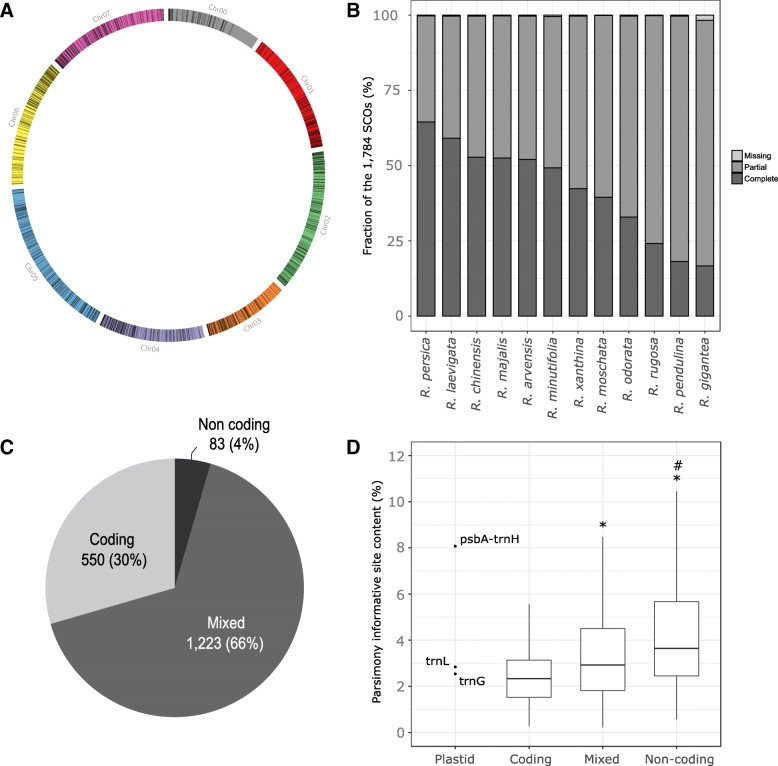
Characterization of the plastid loci and nuclear SCO_Tag_s. **a** Position of the 1784 single-copy orthologs (SCOs) in the seven pseudo chromosomes and unanchored scaffolds (Chr00) of the haploid genome sequence of *Rosa* ‘Old Blush’. **b** Completeness of SCOs in the 12 unassembled rose genomes. *Missing* means that no contig matching the reference SCO could have been assembled; *partial* means that only part of the reference SCO was assembled; *complete* means that the complete reference SCO is covered by at least one assembled contig. **c** Structural annotation of 1856 SCO_Tag_s. **d** Parsimony-informative site (PIS) content for plastid sequences (*psbA-trnH*, *trnL* and *trnG*) and the nuclear SCO_Tag_s. SCO_Tag_s are divided into three categories: coding regions (exons), non-coding (untranslated regions and introns), and mixed regions (containing both coding and non-coding regions). (*) and (#) denote significant differences between coding and mixed regions and between mixed and non-coding regions, respectively (t-test; *p*-value < 0.05)

Using these two sets of single-copy genes, the Reciprocal Best Blast (RBB) procedure identified 1817 shared SCOs between *Rosa* ‘Old Blush’ and the Markov Clustering (mcl) identified 1814 shared SCOs. A total of 1784 SCOs were commonly identified by both methods (Step 2, Fig. 1). These common SCOs are evenly distributed across the seven chromosomes of the haploid genome of *Rosa* ‘Old Blush’ (Fig. 2a). The synteny analysis reveals that the order of SCOs along the genome of *Fragaria vesca* and *R*. ‘Old Blush’ is well conserved (Additional file 1: Figure S1). The great majority (73%) of SCOs that we found are new and were never published before in other ortholog sets (Additional file 1: Figure S2).

### Target assembly and primer design

We applied the automated Target Restricted Assembly Method (aTRAM) for the 1784 selected SCOs to reconstruct (either partly or completely) their corresponding orthologs from the available unassembled genome sequences of 12 *Rosa* species (Table 1). A mean of 1776 SCOs (ranging from 1754 SCOs for *R. gigantea* to 1782 SCOs for *R. moschata*) was retrieved per *Rosa* species (Fig. 2b).

After creating alignments of the aTRAM contigs for each of the 1784 SCOs, we were able to identify 2874 sub-alignments of at least 300 bp that were covered by at least four taxa, including the haploid reference genome of *Rosa* ‘Old Blush’ and the most divergent species *R. persica*. Strict consensus sequences of these sub-alignments were used to design a total of 2339 in silico primer pairs flanking variable non-overlapping tags of 300–550 bp. A total of 1000 out of the 1784 SCOs have at least one tag, with an average of 2.3 tags per SCO (ranging from 1 to 14). Of the 2339 candidate tags, 483 did not pass the post-assembly tests (Step 5, Fig. 1). In details, 46 tags were removed due to unspecific binding of their primer pairs to the haploid reference genome sequence of *Rosa* ‘Old Blush’; 224 tags did not pass the RBB test of orthology; 47 tags did not have a consistent allele number in aTRAM contigs regarding the ploidy level of the unassembled *Rosa* genome; 166 tags did not have a consistent hit number regarding the ploidy level of the *Rosa* genome when BLAST-searched on already assembled *Rosa* datasets. The final set contains 1856 tags that could be used for phylogenomic analyses (Additional file 2). These tags will now be referred to as Single-Copy Orthologous Tags (SCO_Tag_s) in the text, to denote that they are short, PCR-amplifiable sequence tags, derived from primers in conserved sequences that flank variable sequence regions in single-copy orthologous genes identified across a set of closely-related species. Of these 1856 SCO_Tag_s, 1223 (66%) cover both coding and non-coding regions, while 550 (30%) cover pure coding regions and 83 (4%) cover pure non-coding regions (Fig. 2c).

We also searched outgroup species genomes for the presence of the respective 1856 SCO_Tag_s, leading to 1534 SCO_Tag_s that contain at least one of the seven outgroup species (*Fragaria iinumae*: 1029; *F. nipponica*: 875; *F. nubicola*: 858; *F. vesca*: 1142; *Rubus occidentalis*: 985; *Geum urbanum*: 697; *Potentilla micrantha*: 1092). Apart from *Rosa* ‘Old Blush’ and *R. persica*, which are present for all of the 1856 SCO_Tag_s, the taxon occupancy of SCO_Tag_s for the *Rosa* ingroup varies from 23% for *R. palustris* to 97% for *R. wichurana* (Additional file 1: Figure S3). Half of the 1856 SCO_Tag_s have been found in at least 14 out of the 17 *Rosa* species analyzed. Species sequences from each of the 1856 SCO_Tag_s are available in Additional file 3. Species sequences from each SCO_Tag_ were aligned using mafft and cleaned with Gblocks, leading to a supermatrix of 669,354 bp for the ingroup species with 28% of missing data, after the removal of 4843 (0.7%) poorly-aligned sites. For the dataset with ingroup plus outgroup species, the supermatrix contained 676,389 bp with 34% of missing data after the removal of 16,978 (2.4%) poorly-aligned sites.

### Efficiency of plastid loci and nuclear SCO_Tag_s for *Rosa* phylogeny

We analyzed the sequence variation contained in each of the 1856 SCO_Tag_ alignments, focusing only on the *Rosa* ingroup. The mean number of taxa per SCO_Tag_ alignment was 9, 11 and 15 for SCO_Tag_s covering non-coding, mixed and coding regions, respectively. As expected, on average, the non-coding regions contain more parsimony-informative sites (PIS) than mixed sequences, which in turn contain more PIS than pure coding regions (Fig. 2d). Plastid sequences *trnL* and *trnG* have medium PIS content (2–3%), whereas the *psbA-trnH* region is highly variable (> 8% of PIS) and reaches the upper bound of PIS content distributions of both mixed and non-coding sequences (Fig. 2d).

**Fig. 3:**
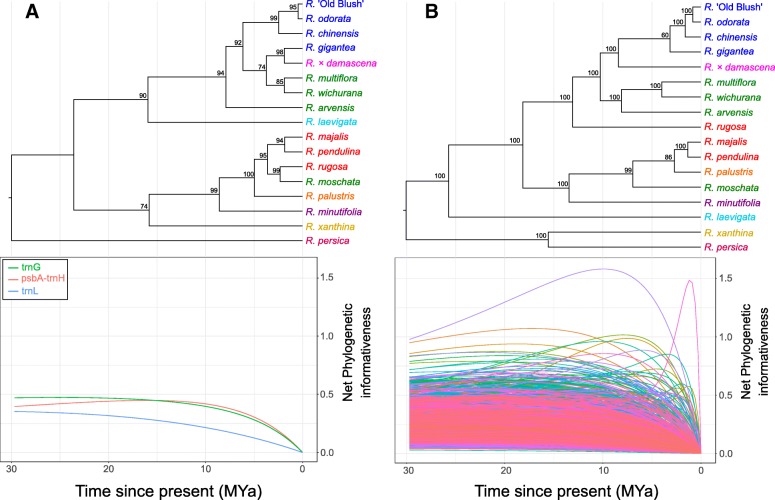
Net phylogenetic informativeness (PI) profiles compared to species chronograms. **a** Plastid loci; **b** 1856 nuclear SCO_Tag_s. Taxa are colored as follows: dark blue for taxa from *Rosa* sect. *Chinenses*, pink for *R*. sect. *Gallicanae*, green for *R*. sect. *Synstylae*, light blue for *R*. sect*. Laevigatae*, red for *R*. sect. *Rosa* (ex. *R*. sect. *Cinnamomeae*), orange for *R*. sect. *Carolinae*, purple for *R*. subg. *Hesperhodos*, yellow for *R*. sect. *Pimpinellifoliae* and fuchsia for *R*. subg. *Hulthemia*

In the nuclear SCO_Tag_ species-chronogram, almost all branches show bootstrap supports (BS) of 100%, in clear contrast with the species-tree obtained based on the conventional plastid sequences (Fig. 3). Both datasets support a distinct *Chinenses*-*Gallicanae*-*Synstylae* clade but have slightly different tree structures for the remaining species. While only the nuclear SCO_Tag_s support monophyly for the *Chinenses* and three of the four *Synstylae*, both datasets exhibit strong support (> 99% BS) for the position of *Rosa moschata* and *R. minutifolia* near the *Rosa* clade. In addition, the nuclear SCO_Tag_s dates the *R. laevigata* speciation event as being more ancient (26 MYa) than the plastid dataset suggests (16 MYa) and supports the monophyly of the two bright yellow-flowered species, *R. persica* and *R. xanthina*.

### Phylogenetic informativeness

The Phylogenetic Informativeness (PI) profiles of the plastid sequences are smooth, with a slow decrease through geological time, and they never reach values above net PI of 0.5 (Fig. 3a). During the last 8 M years, *psbA-trnH* and *trnG* display a similar profile but *trnG* reaches higher PI values for more ancient periods. The *trnL* locus shows lower PI values than the two other plastid loci at all times. The PI profiles of the nuclear SCO_Tag_s have different shapes and heights (Fig. 3b). While most of the SCO_Tag_s do not exceed a net PI of 0.5 during the past 30 M years of divergence, some reach PI values higher than 1.0. A total of 131 SCO_Tag_s reach their maximum value at the 0–15 MYa time interval, which represents the most recent half of the total divergence period and includes 75% of the species-tree nodes. For older nodes, informative SCO_Tag_s can be identified with PI values peaking around 20 MYa with net PI between 0.75 and 1. Additionally, we observed that the area under the PI profiles for the time interval 0–30 MYa tends to decrease while more taxa are added to SCO_Tag_ alignments (y = 11.8–0.45x, *R*^2^ = 0.18). By increasing the number of taxa per alignment from 6 to 17, the average area under the PI profile decreases by a factor of 2 (Additional file 1: Figure S4A). Albeit less clear, the fraction of variable sites in SCO_Tag_ alignments also tends to be negatively correlated with the number of taxa included per SCO_Tag_ alignment, especially for SCO_Tag_ with high taxon occupancy (y = 22.1–0.90x, *R*^2^ = 0.11) (Additional file 1: Figure S4B).

### Analysis of topological conflict

**Fig. 4:**
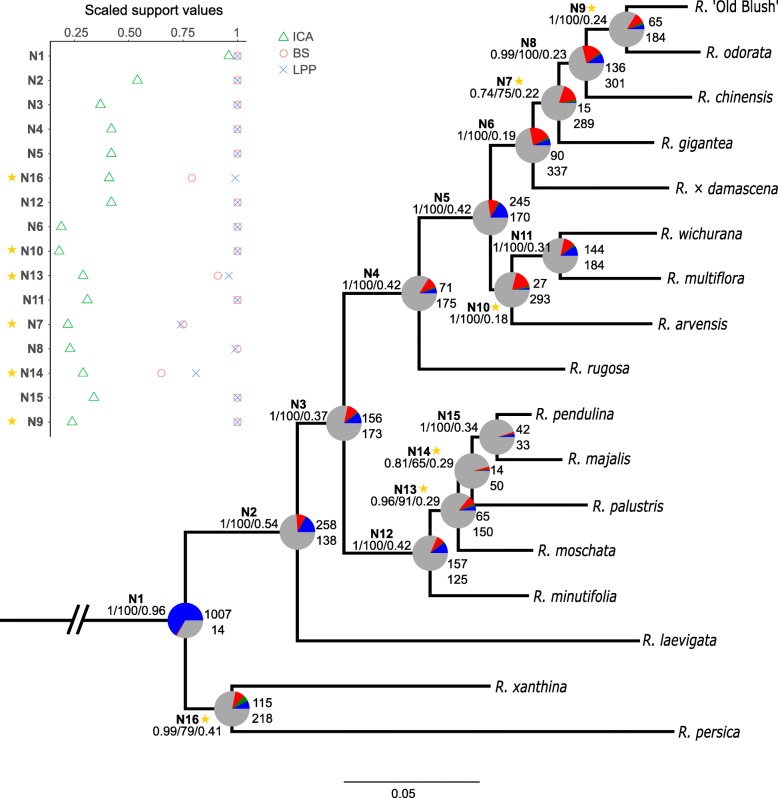
Combined ML species tree with summary of conflicting and concordant SCO_Tag_s. The ML species-tree was constructed from 1526 concatenated rooted SCO_Tag_s. Outgroups are not shown. Node names are in bold. For each branch, the three values separated by a slash are the local posterior probability (LPP), the bootstrap support (BS) and the Internode Certainty All (ICA), respectively. The pie charts at each node present the fraction of SCO_Tag_s that supports that bipartition (blue), the fraction that supports the main alternative bipartition (green), the fraction that supports other alternative bipartitions (red) and the fraction with either less than 70% BS at this bipartition or that do not have this partition due to missing data (gray). On the right side of the pie charts, the top and bottom values indicate the numbers of SCO_Tag_s concordant, respectively in conflict, with the corresponding bipartition in the species-tree. Scatter plot on the left side compares values of BS, LPP and ICA at each node. Nodes are ranked from the most ancient (N1) to the most recent (N9) according to Fig. 3b. Stars indicate conflicting nodes with great fractions of alternative bipartitions

Higher PI profiles of nuclear SCO_Tag_s at a time interval do not necessary correspond to better support values in the corresponding species-chronogram. This is because PI does not directly account for phylogenetic noise [50], so that genes with fast-evolving sites may display high PI profiles, whereas they can increase the number of homoplastic sites and obscure the number of synapomorphic sites which therefore scrambles the phylogenetic signal and provides poor support for bipartitions [76]. Therefore, we also tested our SCO_Tag_s based on topological criteria to ensure that highly informative SCO_Tag_s are concordant with the species-tree and do not result from regions with too many fast evolving sites. We first constructed a network to summarize conflicts between all SCO_Tag_s trees (Additional file 1: Figure S5). Species groups identified in the network are mostly consistent with the clades found in the concatenated analysis (Fig. 3b). The reticulation pattern show conflict between SCO_Tag_ trees for both recent and ancient speciations. For recent speciations, links between species are short and packed while they are long and slack for more ancient speciations. Then, we detailed these conflicts for each node of the species-tree using PhyParts. Of the 1534 SCO_Tag_s with at least one outgroup sequence, 8 did not resolve the monophyly of outgroup species and were therefore discarded since rooted SCO_Tag_ trees are required to detail the underlying conflict at each node of the species-tree. The Maximum Likelihood (ML) species-tree obtained after concatenation of the 1526 resulting SCO_Tag_s is presented in Fig. 4. The topology is the same as for (1) the coalescent species-tree obtained after the reconciliation of the 1526 SCO_Tag_ trees and (2) the chronogram presented in Fig. 3b, but with slight modification of BS for node 7 (increase from 60 to 75%), node 13 (decrease from 99 to 91%), node 14 (decrease from 86 to 65%), node 16 (decrease from 100 to 79%). In addition to BS, we computed two other support values: (1) Local Posterior Probabilities (LPP) that derive from frequencies of quadripartitions observed in the set of SCO_Tag_ trees and (2) Internode Certainty All (ICA) scores that provide information on the amount of conflict at each node. Although not directly related, these three support values each explain in their own way the phylogenetic signal present in the dataset. We observe that low LPP generally correspond to less supported branches (BS < 100%), except for node 16. However, we often observe that high LPP and BS value do not always correspond to high ICA scores (Node 6, 8, 9 and 10). The normalized quartet score for the coalescent tree is 0.73, meaning that 73% of all the quadripartitions found in SCO_Tag_ trees satisfy the coalescent species-tree. We then deconstructed each SCO_Tag_ tree topology and focused only on bipartitions showing > 70% BS that we compared to the bipartitions found in the ML species tree. SCO_Tag_s resolve more bipartitions with a BS > 70% at ancient nodes than at recent nodes. This observation holds as well for the ICA score where the most ancient nodes have higher ICA values than the most recent nodes (Fig. 4). For very recent nodes, few SCO_Tag_s can individually make the distinction between closely related taxa at this BS threshold.

**Fig. 5:**
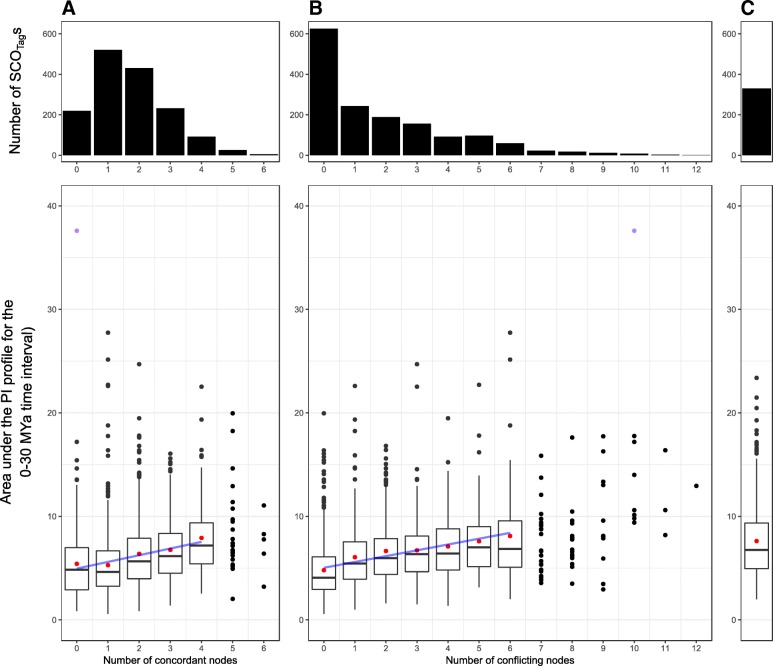
Correlation between phylogenetic informativeness (PI) and the number of **a** concordant nodes and **b** conflicting nodes in SCOTag topologies. **c** corresponds to the PI distribution for unrootable SCOTag that were not analyzed using PhyParts. Situations with less than 30 points were ploted but not used in the calculation of correlations. Red dots correspond to mean values. Blue lines correspond to regression lines: y = 4.95 + 0.65x, *R*^2^ = 0.04 in panel (**a**) and y = 5.03 + 0.56x, *R*^2^ = 0.10 in panel (**b**). The top most purple dot corresponds to the highest PI profile in Fig. 3b

Regarding patterns of concordance and conflict, we first observe that no SCO_Tag_s are concordant with more than six of the 16 nodes present in the species-tree (Fig. 5a), whereas some SCO_Tag_s are in conflict with up to 12 nodes (Fig. 5b). The highly conflicting SCO_Tag_s (conflicting in more than seven nodes) represent a minority (4%) of the entire dataset. Actually, 625 SCO_Tag_s bear 0 conflicting nodes and 1184 SCO_Tag_s agree with one to three nodes. Then, we analyzed the pattern of conflict node by node. We observed that more than two-thirds of the SCO_Tag_s agree in dividing the genus at node 1 with the two yellow-flowered species *Rosa persica* and *R. xanthina* separate from the rest of the *Rosa* species. For more recent nodes, higher number of individual alternative bipartitions can be observed (Fig. 4). Nodes 7, 9, 10, 13, 14 and 16 show a significant proportion of SCO_Tag_s agreeing with the main alternative bipartition, meaning that the proportion of SCO_Tag_s supporting the main alternative bipartition is greater than 50% of the proportion of SCO_Tag_s agreeing with the species-tree bipartition (Additional file 1: Figure S6). These conflicting nodes do not always correspond to the lowest BS, ICA or LPP support values.

### Correlation between phylogenetic informativeness and topological conflict

We then correlated the area under the PI profile for the 0–30 Mya time interval with the number of nodes in SCO_Tag_ tree that are concordant or in conflict with the species-tree, using a BS cutoff of 70% (Fig. 5). We observed that PI tends to increase while more concordant nodes are present in SCO_Tag_ trees (y = 4.95 + 0.65x, *R*^2^ = 0.04). A similar observation can be made for the number of conflicting nodes (y = 5.03 + 0.56x, *R*^2^ = 0.10). Interestingly, we observed that the top most informative SCO_Tag_ identified in Fig. 3b is in fact conflicting in 10 nodes and agrees with 0 node (Fig. 5). In addition, the 330 SCO_Tag_s that have not been analyzed for topological concordance due to lack of outgroups tend to show a similar PI distribution to SCO_Tag_s that were analyzed for topological conflict (Fig. 5c).

Metrics regarding variability content, phylogenetic informativeness and topological conflict for the 1856 SCO_Tag_s are available in Additional file 4.

## Discussion

### Finding nuclear SCO_Tag_s at the genus level

Several sets of SCOs have recently been released but few studies have focused on developing SCOs dedicated to species-level phylogeny [77, 78]. For genera such as *Rosa*, which shows rapid radiations [17, 79], it is likely that DNA sequences (either nuclear or plastid) are very closely-related, and SCOs designed for reconstructing the broad angiosperm phylogeny may not be suited to resolving species relationships. In this study, we took the woodland strawberry (*Fragaria vesca*) as an outgroup to identify SCOs shared with the genus *Rosa*. The two taxa share similar genome characteristics such as diploidy and a base chromosome number of seven. Macro-synteny analysis also revealed only one major translocation event between two chromosomes [67]. In addition, *Fragaria vesca* and *Rosa* species belong to sister tribes within the subfamily *Rosoideae* [80]. The number of single-copy genes that we identified in each of the two species was consistent with previous observations across angiosperms [24]. *Rosa* ‘Old Blush’ is currently the only *Rosa* taxon with a high-quality annotated genome sequence and we chose it as the reference for the whole *Rosa* genus [67]. We identified 1784 conserved genes in the subfamily *Rosoideae* by searching for shared SCOs between *Fragaria vesca* and *Rosa* ‘Old Blush’. We observed a relative shared synteny in the localization of the 1784 SCOs between *F. vesca* and *R*. ‘Old Blush’ which emphasizes on the fact that we selected conserved genes. We also found that 73% of the 1784 SCOs are not present in other published ortholog sets (Additional file 1: Figure S2), suggesting that it is worth developing specific phylogenomic markers that are dedicated to each particular taxonomic group. Then, we considered that the 1784 SCOs identified in *R*. ‘Old Blush’ are also orthologous in other *Rosa* species. We therefore assumed that no more gene duplication or gene loss occurred in the SCO set after the divergence of the *Potentillae* and the *Roseae* tribes around 60 MYa [80]. No recent large genome duplication was detected in *Rosa* ‘Old Blush’ [67]. Since *Rosa* ‘Old Blush’ is considered to be an interspecific hybrid between *R. odorata* and *R. chinensis* [68], two species sharing their last common ancestor some 8–9 MYa [51], this suggests that gene gain by large duplication is not common in closely-related roses. However, our assumption may not hold if fine-scale genome rearrangements occurred in other *Rosa* species that were not analyzed here. This means that paralogous genes might be targeted using our 1784 SCOs on a broader set of *Rosa* species. For this reason, we carried out additional filtering on the tags obtained after the target assembly of the 1784 SCOs. This filtering procedure aimed to eliminate putative paralogous sequences by discarding (1) tags with unspecific primer pairs (Step 5a, Fig. 1), (2) tags that do not have a strict 1-to-1 orthologous relationship with the reference genome of *R*. ‘Old Blush’ (Step 5b, Fig. 1) and (3) tags with an inconsistent number of alleles in either aTRAM contigs (Step 5c, Fig. 1) or already assembled *Rosa* genomes (Step 5d, Fig. 1). Our final set of 1856 SCO_Tag_s derived from 1784 SCOs should therefore essentially contain orthologous sequences suited to phylogenomics analyses.

Using shotgun sequencing libraries and Illumina short-read sequencing at low depth (10-30x) in 12 *Rosa* species, we applied the aTRAM pipeline to assemble specific loci [81], and we retrieved most of the 1784 SCOs (Fig. 2b). While this method does not take individual heterozygosity at each SCO_Tag_ into account, it provides a fast and easy way to extract genome sequences of specific loci, while circumventing whole genome assemblies, which may be particularly difficult for highly heterozygous taxa such as *Rosa* species. Our procedure only retains one representative SCO_Tag_ sequence per species, which may be sufficient for genus section comparisons. However, phylogenomic analyses below the section level may require to reconstruct multiple sequence variants per species to reveal hybrid specimens. For this reason, we developed conserved SCO_Tag_ primer pairs that can be used to target SCO_Tag_ alleles using basic PCR amplifications in future analyses (Additional file 2).

### Efficiency of nuclear SCO_Tag_s for phylogenomics in the genus *Rosa*

We have built a ML phylogenomic tree of some representative species of the genus *Rosa* using 1856 SCO_Tag_s within 1784 SCOs (Fig. 3b), leading to a highly supported species-tree structure. Both plastid loci and nuclear SCO_Tag_s revealed a *Chinenses*-*Gallicanae*-*Synstylae* clade, but only the nuclear SCO_Tag_s supports the monophyly of these three groups within the clade. On the contrary, the plastid loci better resolve the monophyly of the *Rosa* sect. *Rosa* clade, while *R. rugosa* is separated from the rest of *Rosa* sect. *Rosa* species, and found near the *Chinenses*-*Gallicanae*-*Synstylae* clade in the nuclear SCO_Tag_ topology. Compared to previous studies [51, 63], both plastid and nuclear sequences expressed unexpected positions of *R. moschata*, which was expected to group together with the other *Synstylae*, and *R. minutifolia* that was expected to branch off earlier in the phylogenetic tree. These discrepancies may arise from the taxon sampling itself. *R. moschata* and *R. rugosa* have been extensively used in breeding [82] and Hibrand Saint-Oyant et al. [67] may have sampled one of many varieties that were derived from hybridization. The wild origin of *Rosa moschata* is uncertain [83] since several moschata-type roses share a similar geographical distribution from Southeast Europe to the Himalayas, such as *R. beggeriana* Schrenk ex Fisch. & C. A. Meyer, *R. fedtschenkoana* Regel and *R. brunonii* Lindl. [84]. The latter is often cultivated as *R. moschata* in rose gardens [52]. We suggest that the *R. moschata* that we used could be a hybrid between several wild species sharing a common distribution, with at least one species (*R. beggeriana*) belonging to *R*. sect. *Rosa*, the same section as *R. rugosa*. This could explain that *R. moschata* is closely related to the *R*. section *Rosa* in our analysis (Fig. 3). Based on a comparison between plastid loci and nuclear SCO_Tag_s phylogenies, our data may suggest that the maternal origin of our *R. rugosa* is from *R*. sect. *Rosa*, whereas its nuclear genome shows proximity with species of *R*. sect. *Synstylae*, also native to Northeast Asia. This demonstrates the utility of combining plastid and nuclear sequences for phylogenomic analyses to reveal putative hybridization events. The *R. minutifolia* we analyzed here is a white variety known as *R. minutifolia* ‘Alba’, and the accession used shows unexpected morphological characteristics (leaflet size > 3 cm, long pinnate leaves and multi-flowered inflorescences), suggesting an earlier cross with a species from *Rosa* subg. *Rosa*. The ease for *Rosa* species to hybridize poses a major challenge for correct taxonomic identification. This highlights the importance for future studies to preferentially sample several specimens per species, including wild accessions and garden-grown accessions derived from cuttings with a known wild origin.

We further evaluated which of the 1856 SCO_Tag_s performed best for a future phylogenomics study on a broader set of wild species in the *Rosa* genus. PI analyses showed that a large fraction of nuclear SCO_Tag_s have little information content to reconstruct speciation events in the genus *Rosa* with profiles lower than 0.5 of net PI and a slow decrease over time (Fig. 3a). However, a few hundred SCO_Tag_s exhibit high PI profiles that peaked at different ages of the chronogram. Such a diversity of PI profiles is interesting since different sets of SCO_Tag_s could resolve specific levels of the species-tree. Many of the ancient nodes are not well supported in recently published plastid phylogenies of the genus *Rosa* [51, 63] and it would be interesting to target SCO_Tag_s with high PI during ancient evolutionary time intervals. PI profiles of conventional plastid sequences show their limitations to resolve nodes in *Rosa* phylogeny, even for *psbA-trnH* (Fig. 3a) that has a relatively high PIS content, in line with previous works that compared phylogenetic informativeness of nuclear vs. plastid sequences in other groups [77].

We then focused on topological conflict between each SCO_Tag_ tree toward the species-tree (Fig. 4). We mainly show that most SCO_Tag_s cannot individually resolve shallow to intermediate nodes with a BS threshold of 70%. One of the main reasons may be the alignment length of each SCO_Tag_ which is very short and barely exceeds 500 bp. It may therefore be difficult to have enough variable sites for a good confidence in bipartitions within only one SCO_Tag_, especially for recent times where DNA sequences among closely-related taxa are expected to be very similar. SCO_Tag_s that display bipartitions with a BS > 70% often support alternative bipartitions that do not reflect the species-tree. These discrepancies between gene-trees and the species-tree were already observed in other studies [33, 85]. Global patterns of conflict were first summarized on a network (Additional file 1: Figure S5) and further detailed node by node. We observed that the species network highlighted many conflicts between SCO_Tag_ trees although the species groups identified were consistent with the ML species tree. Recent divergences were more prone to conflict as observed with the tight links between close-related species on the network and further confirmed by the decrease of ICA scores for recent nodes (Fig. 4). In details, several nodes showed a high proportion of the main alternative bipartition (Additional file 1: Figure S6). Most of them concern rearrangements between species inside a section clade or between neighboring species that belong to sister clades in Fig. 3b. Conflicts observed at node 7 and node 9 relate to switches between species that belong to the *Chinenses*-*Gallicanae* clade. For instance, the main alternative bipartition found for node 9 involves the switches between *Rosa odorata*, *R. gigantea* and *R. chinensis* as the species that are the most closely related to *Rosa* ‘Old Blush’. Those tree structures can be explained since *R. chinensis* and *R. odorata* va*r. gigantea* are probably the parents of *Rosa* ‘Old Blush’ [68]. The reference genome sequence of *Rosa* ‘Old Blush’ was obtained from a haploid cell line derived from pollen cells [67]. The resulting chromosome set may have contained unequal contributions from the ancestral *R. odorata* and *R. chinensis* genomes after the random meiotic division. Conflicts at node 10 comes from the switch between *Synstylae* species and *Chinenses*-*Gallicanae* species, showing the close relationships between those sections. The dubious positioning of *R. minutifolia* brings conflicts at node 13 and 14 since *R. minutifolia* is found sometimes closer to *R. pendulina* (*R*. sect. *Rosa*), sometimes closer to *R. moschata* (*R*. sect. *Synstylae*), highlighting again the issue of correct taxonomic identification of this accession. Finally, the most ancient node with a significant main alternative bipartition is node 16 and relates to the split of the clade {*R. xanthina*, *R. persica*} into two separate lineages. Despite their bright yellow petals, *R. persica* and *R. xanthina* are very different wild rose species in terms of shapes, habitats and morphological traits [52, 83]. Sampling additional rose species in *Rosa* sect. *Pimpinellifoliae* will be useful in future studies to resolve how these species are related.

### Impact of missing data and topological conflict in SCO_Tag_s selection

In this study, we had to deal with missing or partial data for almost all of the 1784 SCOs (Fig. 2b) and therefore for almost all of the 1856 resulting SCO_Tag_s (Additional file 1: Figure S3). Since the approach to SCO_Tag_ identification involves primer design in strictly conserved sequences flanking variable regions, we only kept SCO alignments covered by at least four taxa, including the reference genome sequence of *Rosa* ‘Old Blush’ and the highly divergent species *Rosa persica*. The variation in the number of species included in the 1784 respective SCO alignments has several underlying reasons and has associated consequences for downstream analysis. The underlying reasons for missing species from SCO alignments may reflect: (1) the actual gene duplication or gene loss in the genome of a given species; (2) insufficient read depth or inability to reconstruct the locus from the whole genome shotgun sequencing data; (3) strong sequence divergence that hampers the recognition of high confidence BLAST identification of orthologous genes from a given species. Furthermore, selecting informative SCO_Tag_s depends on the complex relationship between the number of taxa compared, their sequence divergence (which, in turn, depends on coding/non-coding capacity) and parsimony-informative site (PIS) content. For instance, the more taxa that are compared and the more divergent the species that are included in the alignment are, the more likely it is that variable sites will become parsimony informative, but the less likely it is to identify flanking, strictly conserved regions for primer design. Indeed, classification of the coding potential of SCO_Tag_s based on positional overlap with structural gene model annotation revealed, as expected, that non-coding SCO_Tag_ alignments comprise two-fold less species than pure coding SCO_Tag_ alignments, in line with elevated sequence divergence in non-coding regions compared to protein coding sequences. As a consequence, SCO_Tag_s that contain strictly non-coding regions comprise only 4% of the entire SCO_Tag_ set, and while they contain lower numbers of taxa per alignment, they still exhibit the highest relative PIS content (Fig. 2d). A substantial fraction of our SCO_Tag_s contains both coding and non-coding regions, and selecting this type of SCO_Tag_ may be a good strategy to target conserved regions surrounding variable sequences. By increasing the relative fraction of non-coding SCO_Tag_s, the procedure proposed here may be more informative than exon capture or phylotranscriptomics to decipher phylogenetic relationships for closely-related species or those with complex evolutionary relationships.

Furthermore, we analyzed our set of SCO_Tag_s for phylogenomic informativeness as a function of divergence time as well as for topological conflict. We observed lower PI values for SCO_Tag_s containing the most taxa (Additional file 1: Figure S4A), suggesting that well-covered SCO_Tag_s would not be preferentially sampled based on the PI profile criteria. Klopfstein et al. [76] claim that adding more taxa to the alignment reduces the probability to observe a never-reversed synapomorphy since each new taxon may reverse the synapomorphy and thus lower the optimum evolutionary rate. In contrast, Townsend and Leuenberger [86] argued that increasing taxon sampling does not decrease that optimal rate of character change. Here, all SCO_Tag_ alignments contain sequences of the most divergent wild rose species and at least two other intermediate species. It is therefore unlikely that some loci disproportionally represent ancient vs. recent divergences. We also observed that SCO_Tag_s with few taxa tend to have greater relative numbers of variable sites (Additional file 1: Figure S4B), which may be due to the fact that SCO_Tag_s with less taxon occupancy are less conserved and therefore more variable.

Townsend’s PI does not directly account for noise that may be caused by fast-evolving sites. However, a thorough analysis of PI curves can provide insight into how much noise is present in each SCO_Tag_. Sharp recent peaks with a steep post-slope may introduce noise for older nodes. Consequently, for a given value of PI_max_, it is better to select SCO_Tag_s that express a steady decline after they peak [86, 87]. Despite we did not observed a general strong correlation between PI and topological conflict, we noticed that the top most informative SCO_Tag_ for the 0–30 MYa time interval (Fig. 3b) is also a highly conflicting SCO_Tag_ (Fig. 5b). This demonstrates the importance to combine different approaches to evaluate the set of sequences prior to phylogenomics inferences. This assessment enables to identify the most phylogenetic informative sequences and to reveal patterns of conflicts while a basic supermatrix approach simply conceals conflicts and can even produces a well-supported but incorrect species tree [88, 89]. Atypical SCO_Tag_ should not necessary be removed for downstream phylogenomic analyses since they hold different evolutionary histories that may be interesting to study. Regarding phylogenomics in the genus *Rosa*, the many patterns of conflict, that we especially observed in close-related species, highlight the difficulty to clearly identify one overall evolutionary history in this genus. Patterns of conflicts will have to be taken into account in future studies to accurately unravel the complex mechanisms that shaped this genus. It is also worth mentioning that our sampling covers only one-tenth of the existing wild rose species, and some recent rapidly evolving sections such as *Rosa* sect. *Caninae* are not represented. Thus, we recommend selecting well-covered SCO_Tag_s, that peak at various times during the 30 M years of divergence for future studies on *Rosa* relationships. Using sets of SCO_Tag_s with similar PI values, SCO_Tag_s with maximal numbers of species should be prioritized to increase the chance of successful target PCR amplification.

## Conclusion

The method implemented here to mine genome-scale sequencing data successfully recovered hundreds of nuclear single-copy orthologous sequence tags suitable for species-level phylogenomics in the highly complex genus *Rosa*. We emphasize that a thorough analysis must be performed on phylogenomic datasets in order to choose the most informative markers. While the sequence content of variable sites is obviously important, it does not predict better topology resolution. Computing phylogenetic informativeness and topological conflict of SCO_Tag_s ensures the selection of a comprehensive set of SCO_Tag_s containing appropriate sequence variations to cover the entire period of species divergence and simultaneously reveals potential sources of topological conflict that may have biological meanings, such as hybridization events or unwanted selection of paralogous copies. Despite the fact that plastid sequences are less variable, their one-sided inheritance still gives valuable perspectives for comparison with nuclear data in view of a better understanding of how evolutionary processes, such as hybridization, shape complex genera such as *Rosa*. The mining strategy presented here enables the development of SCO_Tag_ nuclear markers to target yet unresolved parts of the green plants’ Tree of Life, from the deepest branches to the shallowest relationships between individuals.

## Methods

### Identification of single-copy orthologs in *Rosa* ‘Old Blush’ and *Fragaria vesca*

Single-copy nuclear genes were identified by comparing annotated protein sets in the haploid reference genome sequences of *Rosa* ‘Old Blush’ [67] and *Fragaria vesca* [71]. First, the annotated protein set from each genome was compared to itself using an all-against-all BLAST+ [90] search. Outputs were parsed using the tcl script [91] with an e-value cutoff of 1e-10, identity of at least 30% and coverage above 70% of the query. Single-copy nuclear genes were identified as those with a unique blast hit to themselves (Step 1, Fig. 1). Next, two methods were used to identify single-copy orthologs (SCOs) shared between *Rosa* ‘Old Blush’ and *F. vesca*. In the first method, a reciprocal best-hit blast (RBB) was performed between *Rosa* ‘Old Blush’ and *F. vesca* sets of single-copy genes, and SCOs were identified as pairs of proteins with each other as the best scoring match in the respective genome. Second, the Markov clustering algorithm (mcl) method [92] was run via the mclblastline command [93] to cluster all single-copy proteins from *Rosa* ‘Old Blush’ and *F. vesca* into groups using an inflation value of intermediate stringency (3.0). Genes found as SCOs in both methods were retained for downstream analysis (Step 2, Fig. 1). A synteny analysis was also performed to compare the position of the SCOs in the genome assemblies of *F. vesca* [94] and *R*. ‘Old Blush’, and to further assess the orthology assumption. Finally, we also used BLAST with the above settings to compare our set of SCOs to three published ortholog sets to evaluate the redundancy of our SCOs (957 *Arabidopsis-Populus-Vitis-Oryza* (APVO) single-copy genes [23], 257 Low-Copy Nuclear Genes for *Rosaceae* phylogenomics (LCNG) [27] and 1041 *Rosaceae* Conserved Ortholog Set of markers (RosCOS) [26]).

### Reconstruction of nuclear SCOs and plastid loci in *Rosa* sp.

To identify sequence variations within the SCOs across the genus *Rosa*, we retrieved the corresponding sequences from already published whole genome shotgun (WGS) Illumina paired-end sequence data of 16 *Rosa* species (Table 1 and Fig. 1). For 12 unassembled genomes, WGS reads were processed with the aTRAM v1.0 iterative pipeline [81] to assemble the SCOs. Briefly, reads are first assigned to partitions, also called shards, to ease the pipeline parallelization and to optimize the computing needs. Second, a SCO protein sequence is used as a query to retrieve homologous reads through a BLASTX search against shards. Corresponding forward or reverse reads are then retrieved for ABySS v2.0 assembly [95]. Assembled contigs are iteratively used as queries for the next round of assembly. As a result, contig length increases and this iterative process may lead to the assembly of the entire SCO locus, including introns and untranslated regions. We performed three iterations of assembly on the GenoToul bioinformatics high-performance computing cluster using 16 cores of Intel® Xeon® computers with a 2.50GHz processor. For each SCO in each unassembled *Rosa* genome, the contig with the highest alignment score on the *Rosa* ‘Old Blush’ reference SCO sequence was selected as the representative orthologous sequence for this genome (Step 3, Fig. 1). Then, for each SCO, we created mafft [96] alignments between all orthologous sequences. Alignments were screened to find regions covered by at least four taxa, including *Rosa* ‘Old Blush’ and *Rosa persica*, considered as the most divergent *Rosa* taxon [51] and even considered to be in a separate genus in former classifications [97, 98]. Strict consensus sequences of these regions were used to design generic conserved primer pairs with Primer3 [99], so that any fragment could further be amplified using the polymerase chain reaction technique (Step 4, Fig. 1). Conditions for primer design were a melting temperature between 59 °C and 61 °C, a maximal homo-polymer of 3, at least one 3′-GC clamp and amplicon size between 300 bp and 550 bp. For each SCO, a maximum of 100 primer pairs spanning the entire consensus sequence were designed. We then selected the most variable non-overlapping amplicon tags for each region and checked for specificity of their corresponding primers on the haploid reference genome of *Rosa* ‘Old Blush’ (Step 5a, Fig. 1). We additionally retrieved positional information on untranslated transcribed region (UTR), intron, and exon locations for each tag. To further assess the orthology assumption of the tags, we ran additional tests. First, we checked that each tag has a number of alleles in assembled aTRAM contigs that is compatible with the species genome ploidy level (Step 5b, Fig. 1). Then, we subjected each targeted tag sequence to a reciprocal-best BLAST (Step 5c, Fig. 1). Since the sequence of *R*. ‘Old Blush’ was used as the query for the aTRAM assembly, blasting each targeted tag sequence back to the genome of *R*. ‘Old Blush’ provide the RBB test for orthology. Any tag sequence that did not pass these two tests led to the rejection of all *Rosa* sequences associated with this tag for downstream analysis. Finally, the corresponding tag were retrieved from recently assembled genomes of three additional *Rosa* species (Table 1) (Step 5d, Fig. 1). We also used an assembled transcriptome of *Rosa palustris* [70] because it belongs to the *Rosa* sect. *Carolinae* and is related to several wild roses native to North America. Only exonic tag can be retrieved from transcriptome sequencing data of *R. palustris*. We used a BLAST search to retrieve tag sequences from assembled genomes/transcriptome of *Rosa* species (e-value ≤1e-10; identity ≥65%; 100% coverage of the consensus query tag; maximum query-subject length difference of ±20%). If multiple best hits were found, we arbitrary choose one of them as the representing sequence for the *Rosa* species. If the number of best hits was not consistent with the ploidy level of the *Rosa* species genome, we discarded all sequences related to this tag for downstream analysis. We additionally checked that edges of retrieved sequences corresponded to primer pairs. Thanks to the different filtering procedures that we applied on the initial set of tags, we considered that the resulting tags are Single-Copy Orthologous tags (SCO_Tag_s), suited to reconstructing phylogenomics relationships in the genus *Rosa*. We applied the same procedure as Step 5d, Fig. 1 to identify similar SCO_Tag_ sequences in seven sister outgroups belonging to the subfamily *Rosoideae* (*Rubus occidentalis*, *Fragaria vesca*, *Fragaria iinumae*, *Fragaria nipponica*, *Fragaria nubicola*, *Geum urbanum*, and *Potentilla micrantha*) (Table 1), except that we did not check that edges of sequences strictly corresponded to the respective primer pairs and that we did not discard all SCO_Tag_ sequences if the number of best hits was not consistent with the ploidy level of the outgroup species genome.

The same procedure was applied to retrieve three plastid sequences (*psbA-trnH*, *trnG* and *trnL*) from (un) assembled genome sequences of the same *Rosa* species. When the procedure failed to assemble plastid sequences, we retrieved corresponding plastid sequences from NCBI GenBank (Additional file 5: Table S1).

### Assessment of phylogenomic utility

SCO_Tag_ sequences from different species were aligned using mafft [96], and Gblock [100] was used to trim poorly aligned regions. Variable and parsimony-informative site (PIS) contents were calculated per SCO_Tag_ alignment. Gaps were treated as a fifth base. We then computed phylogenetic informativeness (PI) per SCO_Tag_ using the formula presented in [50] and implemented in the PhyDesign online application [101]. For this analysis, all SCO_Tag_ alignments were concatenated into a super-matrix and the best partition scheme was searched with PartitionFinder v2.1.1 [102]. The partitioned matrix served to construct a Maximum Likelihood (ML) species-tree using RAxML v8.1.5 [103]. The species-tree was then converted to a chronogram in R using the function *chronos* in the package ape [104] by applying one calibration point on the crown node of *Rosa*, dated at 30 MYa [51]. We uploaded the partition concatenated matrix and the chronogram on PhyDesign. Substitution rates were calculated for each SCO_Tag_ in HyPhy [105] using the best generalized time-reversible (GTR) model, with empirical base frequencies, found for the super-matrix in jModeltest2 [106]. Some SCO_Tag_ alignments have sites for which substitution rate was incorrectly determined leading to high spikes close to time 0. Since those high spikes have no real biological meaning and correspond to artefacts, we decided to remove them. To do so, we first identify SCO_Tag_ with such spikes by looking for SCO_Tag_ PI profiles with more than 1 maximum. Second, we retrieved the estimated substitution rates for each SCO_Tag_ with high spikes and looked for an elbow in the distribution of substitution rates. The substitution rate found at the elbow served as a threshold to discard sites with unusual substitution rate. We repeated this second step one time to totally remove high spikes from PI profiles. The python script (PhantomSpikesRemover.py) that we developed to trim SCO_Tag_ PI profiles and alignments is available at https://github.com/kdebray/SCOtags. The same procedure was applied to the three plastid loci to recover their PIS content and PI profiles.

To further determine the underlying phylogenetic conflicts between SCO_Tag_s, we looked for well-supported incongruences between SCO_Tag_ tree topologies. For each SCO_Tag_ alignment with at least one outgroup sequence, we determined the best nucleotide substitution model using jModelTest v2 [106], and we estimated corresponding ML tree with PhyML [107]. We then used PhyParts [108] to map resulting SCO_Tag_-trees onto the species-tree topology, previously obtained by concatenation of all SCO_Tag_s followed by a ML tree estimation. Briefly, each gene-tree is rooted on outgroup species and then split into bipartitions that are compared to all bipartitions present in the species-tree. A gene-tree bipartition *h* is concordant with a species-tree bipartition *s* if all of the ingroup of *h* is included in the ingroup of *s* and if all of the outgroup of *h* is included in the outgroup of *s* [108]. We applied a bootstrap filter of 70% so that only medium to well-supported bipartitions are taken into account for the concordance calculations. As a result, each node of the species-tree is labeled with the fraction of concordant SCO_Tag_s and conflicting SCO_Tag_s. In addition, we used Astral [109] v5.6.3 with default parameters to build a coalescent species-tree from the SCO_Tag_ trees and to compute Local Posterior Probabilities associated with each quadripartitions of the coalescent species-tree. We also calculated the Internode Certainty All (ICA) for each node of the species-tree topology, as implemented in PhyParts. ICA values near 0 indicate major conflicts with similar frequencies among conflicting bipartitions. ICA values near 1 indicate a strong certainty in the bipartition, meaning that few alternative bipartitions with low frequencies have been found. Although ICA score is not directly comparable to bootstrap support (BS), it provides more information about the distribution of conflicts among phylogenomic loci for a specific bipartition [108]. In addition, we also summarized topological conflict between SCO_Tag_ trees through a species network. For this analysis, we first collapsed branches that were poorly supported (ie. BS < 70%) using a custom R script and the function di2multi in the ape package. Then, we combined all clean SCO_Tag_ trees in a FilteredSuperNetwork as implemented in SplitsTree [110] v4.

## Additional files

Additional file 1 : Supplementary figures. PDF file presenting the six supplementary figures cited in the core article. Each supplementary figure appears on a separate page and has its own caption. (PDF 2363 kb)

Additional file 2 : List of the 1856 SCO_Tag_s primer pairs for *Rosa* phylogenomics. Excel file listing sequences of primer pairs associated with the 1856 SCO_Tag_s, as well as information about melting temperature and corresponding SCO_Tag_ amplicons in the haploid reference genome of *Rosa* ‘Old Blush’ (genome coordinates and fragment length). (XLSX 178 kb)

Additional file 3 : Sequences of the 1856 SCO_Tag_s across seven Rosaceae outgroups and 12 *Rosa* species. Raw fasta sequences associated with the 1856 SCO_Tag_s per species. These sequences correspond to either target-assembled SCO_Tag_s from whole genome shotgun Illumina paired-end reads or SCO_Tag_s that were found in already assembled datasets. (TXT 11170 kb)

Additional file 4 : Metrics associated with the 1856 SCO_Tag_s. Excel file containing all metrics that served to assess the phylogenetic utility of the 1856 SCO_Tag_s. Metrics such as sequence variability, phylogenetic informativeness, node-by-node topological conflict and structural annotation are detailed for each of the 1856 SCO_Tag_s. (XLSX 722 kb)

Additional file 5 : Word file containing the supplementary Table S1. (DOCX 14 kb)

## Data Availability

The 1,856 SCO_Tag_s sequences are available in Additional file 4. The 1,856 primer pairs are available in Additional file 2. All SCO_Tag_ sequences used in this study are available in Additional file 3 and at DOI:10.6084/m9.figshare.7907249. All metrics regarding structural annotation, variability content, topological conflict and phylogenetic informativeness are available in Additional file 4. Some custom scripts developed for this study are available at https://github.com/kdebray/SCOtags. The entire set of *Rosa wichurana* genome assembly scaffolds have been deposited at DDBJ/ENA/GenBank under the accession RQIQ00000000. The version described in this paper is version RQIQ01000000.

## Abbreviations

APVO: *Arabidopsis*-*Populus*-*Vitis*-*Oryza*
aTRAM: Automated target restricted assembly method
BLAST: Basic local alignment search tool
BS: Bootstrap support
GAPDH: Glyceraldehyde 3-phosphate dehydrogenase
GTR: Generalized time-reversible
ICA: Internode certainty all
LCNG: Low-copy nuclear gene
LPP: Local posterior probability
mcl: Markov clustering
ML: Maximum likelihood
MYa: Million years ago
NGS: Next-generation sequencing
nrITS: Nuclear ribosomal internal transcribed spacers
PCR: Polymerase chain reaction
PI: Phylogenetic informativeness
PIS: Parsimony-informative site
RBB: Reciprocal best blast
RosCOS: Rosaceae conserved ortholog set of markers
SCG: Single-copy gene
SCO: Single-copy ortholog
SCO_Tag_: Single-copy orthologous tag
UTR: Untranslated transcribed region
WGS: Whole genome shotgun

## References

[1] Straub SCK, Parks M, Weitemier K, Fishbein M, Cronn RC, Liston A (2012). Navigating the tip of the genomic iceberg: next-generation sequencing for plant systematics. Am J Bot.

[2] Matasci N, Hung L-H, Yan Z, Carpenter EJ, Wickett NJ, Mirarab S (2014). Data access for the 1,000 plants (1KP) project. Gigascience..

[3] Cheng S, Melkonian M, Smith SA, Brockington S, Archibald JM, Delaux P-M (2018). 10KP: a phylodiverse genome sequencing plan. Gigascience..

[4] Stevens PF (2017). Angiosperm phylogeny website, version 14, July 2017 [and more or less continuously updated since].

[5] Soltis D, Soltis P, Endress P, Chase M, Manchester S, Judd W (2018). Phylogeny and evolution of the angiosperms: revised and updated edition.

[6] Refulio-Rodriguez NF, Olmstead RG (2014). Phylogeny of Lamiidae. Am J Bot.

[7] Hughes CE, Eastwood RJ, Bailey CD (2006). From famine to feast? Selecting nuclear DNA sequence loci for plant species-level phylogeny reconstruction. Philos Trans R Soc B Biol Sci.

[8] Lyu J, Song J, Liu Y, Wang Y, Li J, Du FK (2018). Species boundaries between three sympatric oak species: *Quercus aliena*, *Q. dentata*, and *Q. variabilis* at the Northern edge of their distribution in China. Front Plant Sci.

[9] Soltis PS, Soltis DE (2009). The role of hybridization in plant speciation. Annu Rev Plant Biol.

[10] Ren R, Wang H, Guo C, Zhang N, Zeng L, Chen Y (2018). Widespread whole genome duplications contribute to genome complexity and species diversity in angiosperms. Mol Plant.

[11] Mallet J (2007). Hybrid speciation. Nature.

[12] Shaw J, Lickey EB, Beck JT, Farmer SB, Liu W, Miller J (2005). The tortoise and the hare II: relative utility of 21 noncoding chloroplast DNA sequences for phylogenetic analysis. Am J Bot.

[13] Gitzendanner MA, Soltis PS, Wong GK-S, Ruhfel BR, Soltis DE (2018). Plastid phylogenomic analysis of green plants: a billion years of evolutionary history. Am J Bot.

[14] Babineau M, Gagnon E, Bruneau A (2013). Phylogenetic utility of 19 low copy nuclear genes in closely related genera and species of caesalpinioid legumes. South Afr J Bot.

[15] Sang T (2002). Utility of low-copy nuclear gene sequences in plant phylogenetics. Crit Rev Biochem Mol Biol.

[16] Reboud X, Zeyl C (1994). Organelle inheritance in plants. Heredity.

[17] Joly S, Starr JR, Lewis WH, Bruneau A (2006). Polyploid and hybrid evolution in roses east of the Rocky Mountains. Am J Bot.

[18] Canback B, Andersson SGE, Kurland CG (2002). The global phylogeny of glycolytic enzymes. Proc Natl Acad Sci.

[19] Martin WF, Cerff R (2017). Physiology, phylogeny, early evolution, and GAPDH. Protoplasma.

[20] Poczai P, Hyvönen J (2010). Nuclear ribosomal spacer regions in plant phylogenetics: problems and prospects. Mol Biol Rep.

[21] Naumann J, Symmank L, Samain M-S, Müller KF, Neinhuis C, Wanke S (2011). Chasing the hare - evaluating the phylogenetic utility of a nuclear single copy gene region at and below species level within the species rich group *Peperomia* (Piperaceae). BMC Evol Biol.

[22] Li M, Wunder J, Bissoli G, Scarponi E, Gazzani S, Barbaro E (2008). Development of COS genes as universally amplifiable markers for phylogenetic reconstructions of closely related plant species. Cladistics.

[23] Duarte JM, Wall PK, Edger PP, Landherr LL, Ma H, Pires PK (2010). Identification of shared single copy nuclear genes in *Arabidopsis*, *Populus*, *Vitis* and *Oryza* and their phylogenetic utility across various taxonomic levels. BMC Evol Biol.

[24] Han F, Peng Y, Xu L, Xiao P (2014). Identification, characterization, and utilization of single copy genes in 29 angiosperm genomes. BMC Genomics.

[25] Liu M, Zhao J, Wang J, Liu Z, Liu G (2017). Phylogenetic analysis of 25 plant species representing 19 angiosperm families and one gymnosperm family based on 390 orthologous genes. Plant Syst Evol.

[26] Cabrera A, Kozik A, Howad W, Arus P, Iezzoni AF, Knaap E (2009). Development and bin mapping of a Rosaceae Conserved Ortholog Set (COS) of markers. BMC Genomics.

[27] Liston A (2014). 257 nuclear genes for Rosaceae phylogenomics.

[28] Lemmon AR, Lemmon EM (2012). High-throughput identification of informative nuclear loci for shallow-scale phylogenetics and phylogeography. Syst Biol.

[29] Small RL, Cronn RC, Wendel JF (2004). L. A. S. JOHNSON REVIEW no. 2. Use of nuclear genes for phylogeny reconstruction in plants. Aust Syst Bot.

[30] Roure B, Rodriguez-Ezpeleta N, Philippe H (2007). SCaFoS: a tool for selection, concatenation and fusion of sequences for phylogenomics. BMC Evol Biol.

[31] Bleidorn C (2017). Phylogenomics - an introduction.

[32] Anonymous author. Surfing the genomic new wave. Nat Plants. 2018;4:393. 10.1038/s41477-018-0209-7 29977087

[33] Maddison WP, Wiens JJ (1997). Gene trees in species trees. Syst Biol.

[34] Jeffroy O, Brinkmann H, Delsuc F, Philippe H (2006). Phylogenomics: the beginning of incongruence?. Trends Genet.

[35] Prasad AB, Allard MW, Green ED, NISC Comparative Sequencing Program (2008). Confirming the phylogeny of mammals by use of large comparative sequence data sets. Mol Biol Evol.

[36] Degnan JH, Rosenberg NA (2009). Gene tree discordance, phylogenetic inference and the multispecies coalescent. Trends Ecol Evol.

[37] Patané JSL, Martins J, Setubal JC, Setubal JC, Stoye J, Stadler PF (2018). Phylogenomics. Comparative genomics.

[38] Bapteste E, Susko E, Leigh J, MacLeod D, Charlebois R, Doolittle W (2005). Do orthologous gene phylogenies really support tree-thinking?. BMC Evol Biol.

[39] Von Haeseler A (2012). Do we still need supertrees?. BMC Biol.

[40] Robinson DF, Foulds LR (1981). Comparison of phylogenetic trees. Math Biosci.

[41] Kuhner MK, Felsenstein J (1994). A simulation comparison of phylogeny algorithms under equal and unequal evolutionary rates. Mol Biol Evol.

[42] Farris JS, Källersjö M, Kluge AG, Bult C (1995). Testing significance of incongruence. Cladistics.

[43] Huelsenbeck J, Bull JJ (1996). A likelihood ratio test to detect conflicting phylogenetic signal. Syst Biol.

[44] Waddell PJ, Kishino H, Ota R (2000). Rapid evaluation of the phylogenetic congruence of sequence data using likelihood ratio tests. Mol Biol Evol.

[45] Planet PJ, Sarkar IN (2005). mILD: a tool for constructing and analyzing matrices of pairwise phylogenetic character incongruence tests. Bioinformatics.

[46] Leigh JW, Susko E, Baumgartner M, Roger AJ (2008). Testing congruence in phylogenomic analysis. Syst Biol.

[47] Leigh JW, Schliep K, Lopez P, Bapteste E (2011). Let them fall where they may: congruence analysis in massive phylogenetically messy data sets. Mol Biol Evol.

[48] Gori K, Suchan T, Alvarez N, Goldman N, Dessimoz C (2016). Clustering genes of common evolutionary history. Mol Biol Evol.

[49] Narechania A, Baker R, DeSalle R, Mathema B, Kolokotronis S-O, Kreiswirth B (2016). Clusterflock: a flocking algorithm for isolating congruent phylogenomic datasets. Gigascience..

[50] Townsend JP (2007). Profiling phylogenetic informativeness. Syst Biol.

[51] Fougère-Danezan M, Joly S, Bruneau A, Gao X-F, Zhang L-B (2015). Phylogeny and biogeography of wild roses with specific attention to polyploids. Ann Bot.

[52] Rehder A (1940). *Rosa* L. Manual of cultivated trees and shrubs hardy in North America.

[53] Wissemann V, Roberts AV, Debener T, Gudin S (2003). Conventional taxonomy (wild roses). Encyclopedia of Rose science.

[54] Jarvis CE (1992). Seventy-two proposals for the conservation of types of selected Linnaean generic names, the report of subcommittee 3C on the lectotypification of Linnaean generic names. Taxon.

[55] Iwata H, Kato T, Ohno S (2000). Triparental origin of Damask roses. Gene.

[56] Matsumoto S, Kouchi M, Fukui H, Ueda Y (2000). Phylogenetic analyses of the subgenus *Eurosa* using the ITS nrDNA sequence. Acta Hortic.

[57] Matsumoto S, Nishio H, Ueda Y, Fukui H (2000). Phylogenetic analyses of genus *Rosa*: polyphyly of section *Pimpinellifoliae* and origin of *Rosa* × *fortuniana* Lindl. Acta Hortic.

[58] Wu S, Ueda Y, Nishihara S, Matsumoto S (2001). Phylogenetic analysis of Japanese *Rosa* species using DNA sequences of nuclear ribosomal internal trancribed spacers (ITS). J Hortic Sci Biotechnol.

[59] Wissemann V, Ritz CM (2005). The genus *Rosa* (Rosoideae, Rosaceae) revisited: molecular analysis of nrITS-1 and *atp*B-*rbc*L intergenic spacer (IGS) versus conventional taxonomy. Bot J Linn Soc.

[60] Qiu X, Zhang H, Wang Q, Jian H, Yan H, Zhang T (2012). Phylogenetic relationships of wild roses in China based on nrDNA and *mat*K data. Sci Hortic.

[61] Qiu X, Zhang H, Jian H, Zhou N, Yan H, Tang K (2013). Genetic relationships of wild roses, old garden roses, and modern roses based on internal transcribed spacers and *mat*K sequences. Hortscience.

[62] Matsumoto S, Kouchi M, Yabuki J, Kusunoki M, Ueda Y, Fukui H (1998). Phylogenetic analyses of the genus *Rosa* using the *mat*K sequence: molecular evidence for the narrow genetic background of modern roses. Sci Hortic.

[63] Bruneau A, Starr JR, Joly S (2007). Phylogenetic relationships in the genus *Rosa*: new evidence from chloroplast DNA sequences and an appraisal of current knowledge. Syst Bot.

[64] Kellner A, Ritz CM, Wissemann V (2014). Low genetic and morphological differentiation in the European species complex of *Rosa sherardii*, *R*. *mollis* and *R*. *villosa* (*Rosa* section *Caninae* subsection *Vestitae*). Bot J Linn Soc.

[65] Liu C, Wang G, Wang H, Xia T, Zhang S, Wang Q (2015). Phylogenetic relationships in the genus *Rosa* revisited based on *rpl16*, *trnL-F*, and *atpB-rbcL* sequences. Hortscience.

[66] Raymond O, Gouzy J, Just J, Badouin H, Verdenaud M, Lemainque A (2018). The *Rosa* genome provides new insights into the domestication of modern roses. Nat Genet.

[67] Hibrand Saint-Oyant L, Ruttink T, Hamama L, Kirov I, Lakhwani D, Zhou NN (2018). A high-quality genome sequence of *Rosa chinensis* to elucidate ornamental traits. Nat Plants.

[68] Meng J, Fougère-Danezan M, Zhang L-B, Li D-Z, Yi T-S (2011). Untangling the hybrid origin of the Chinese tea roses: evidence from DNA sequences of single-copy nuclear and chloroplast genes. Plant Syst Evol.

[69] Nakamura N, Hirakawa H, Sato S, Otagaki S, Matsumoto S, Tabata S (2017). Genome structure of *Rosa multiflora*, a wild ancestor of cultivated roses. DNA Res.

[70] Johnson MTJ, Carpenter EJ, Tian Z, Bruskiewich R, Burris JN, Carrigan CT (2012). Evaluating methods for isolating total RNA and predicting the success of sequencing phylogenetically diverse plant transcriptomes. PLoS One.

[71] Shulaev V, Sargent DJ, Crowhurst RN, Mockler TC, Folkerts O, Delcher AL (2011). The genome of woodland strawberry (*Fragaria vesca*). Nat Genet.

[72] Hirakawa H, Shirasawa K, Kosugi S, Tashiro K, Nakayama S, Yamada M (2014). Dissection of the octoploid strawberry genome by deep sequencing of the genomes of *Fragaria* species. DNA Res.

[73] Jordan CY, Lohse K, Turner F, Thomson M, Gharbi K, Ennos RA (2018). Maintaining their genetic distance: little evidence for introgression between widely hybridizing species of *Geum* with contrasting mating systems. Mol Ecol.

[74] Buti M, Moretto M, Barghini E, Mascagni F, Natali L, Brilli M (2018). The genome sequence and transcriptome of *Potentilla micrantha* and their comparison to *Fragaria vesca* (the woodland strawberry). Gigascience..

[75] VanBuren R, Bryant D, Bushakra JM, Vining KJ, Edger PP, Rowley ER (2016). The genome of black raspberry (*Rubus occidentalis*). Plant J.

[76] Klopfstein S, Kropf C, Quicke DLJ (2010). An evaluation of phylogenetic informativeness profiles and the molecular phylogeny of Diplazontinae (Hymenoptera, Ichneumonidae). Syst Biol.

[77] Granados Mendoza C, Naumann J, Samain M-S, Goetghebeur P, De Smet Y, Wanke S (2015). A genome-scale mining strategy for recovering novel rapidly-evolving nuclear single-copy genes for addressing shallow-scale phylogenetics in *Hydrangea*. BMC Evol Biol.

[78] Kates HR, Soltis PS, Soltis DE (2017). Evolutionary and domestication history of *Cucurbita* (pumpkin and squash) species inferred from 44 nuclear loci. Mol Phylogenet Evol.

[79] Herklotz V, Ritz CM (2017). Multiple and asymmetrical origin of polyploid dog rose hybrids (*Rosa* L. sect. *Caninae* (DC.) Ser.) involving unreduced gametes. Ann Bot.

[80] Xiang Y, Huang C-H, Hu Y, Wen J, Li S, Yi T (2016). Evolution of Rosaceae fruit types based on nuclear phylogeny in the context of geological times and genome duplication. Mol Biol Evol.

[81] Allen JM, Huang DI, Cronk QC, Johnson KP (2015). aTRAM - automated target restricted assembly method: a fast method for assembling loci across divergent taxa from next-generation sequencing data. BMC Bioinformatics.

[82] Wylie A (1954). The history of garden roses, part 1. J R Hortic Soc.

[83] Masure P (2013). Guide des rosiers sauvages: 500 espèces, variétés et hybrides du monde.

[84] Schramm D gh (2016). Damask roses: an untold story. Rose Lett.

[85] Nichols Richard (2001). Gene trees and species trees are not the same. Trends in Ecology & Evolution.

[86] Townsend JP, Leuenberger C (2011). Taxon sampling and the optimal rates of evolution for phylogenetic inference. Syst Biol.

[87] Hilu KW, Black CM, Oza D (2014). Impact of gene molecular evolution on phylogenetic reconstruction: a case study in the Rosids (superorder Rosanae, angiosperms). PLoS One.

[88] Salichos L, Rokas A (2013). Inferring ancient divergences requires genes with strong phylogenetic signals. Nature.

[89] Kubatko L, Degnan JH (2007). Inconsistency of phylogenetic estimates from concatenated data under coalescence. Syst Biol.

[90] Camacho C, Coulouris G, Avagyan V, Ma N, Papadopoulos J, Bealer K (2009). BLAST+: architecture and applications. BMC Bioinformatics.

[91] Kozik A, Chan B, Michelmore R. Tcl/Tk NCBI BLAST PARSER. tclsh: University of California, Davis; 2005. http://cgpdb.ucdavis.edu/BlastParser/Blast_Parser.html.

[92] Enright AJ, Dongen SV, Ouzounis CA (2002). An efficient algorithm for large-scale detection of protein families. Nucleic Acids Res.

[93] van Dongen S (2012). mclblastline - a pipeline for clustering from BLAST files.

[94] Edger PP, VanBuren R, Colle M, Poorten TJ, Wai CM, Niederhuth CE, et al. Single-molecule sequencing and optical mapping yields an improved genome of woodland strawberry (*Fragaria vesca*) with chromosome-scale contiguity. Gigascience. 2018;7. 10.1093/gigascience/gix124. 10.1093/gigascience/gix124 PMC5801600 29253147

[95] Jackman SD, Vandervalk BP, Mohamadi H, Chu J, Yeo S, Hammond SA (2017). ABySS 2.0: resource-efficient assembly of large genomes using a bloom filter. Genome Res.

[96] Katoh K, Standley DM (2013). MAFFT multiple sequence alignment software version 7: improvements in performance and usability. Mol Biol Evol.

[97] Dumortier BC (1824). Notice sur un nouveau genre de plantes: *Hulthemia*; précédée d’un aperçu sur la classification des roses.

[98] Robyns W (1938). G. A. Boulenger 1858-1937. Sa vie et son oeuvre rhodologique. Bull Jard Bot LÉtat À Brux.

[99] Untergasser A, Cutcutache I, Koressaar T, Ye J, Faircloth BC, Remm M (2012). Primer3 - new capabilities and interfaces. Nucleic Acids Res.

[100] Castresana J (2000). Selection of conserved blocks from multiple alignments for their use in phylogenetic analysis. Mol Biol Evol.

[101] López-Giráldez F, Townsend JP (2011). PhyDesign: an online application for profiling phylogenetic informativeness. BMC Evol Biol.

[102] Lanfear R, Calcott B, Kainer D, Mayer C, Stamatakis A (2014). Selecting optimal partitioning schemes for phylogenomic datasets. BMC Evol Biol.

[103] Stamatakis A (2014). RAxML version 8: a tool for phylogenetic analysis and post-analysis of large phylogenies. Bioinformatics.

[104] Paradis E, Blomberg S, Bolker B, Brown J, Claude J, Cuong HS (2017). ape: analyses of phylogenetics and evolution.

[105] Pond SLK, Frost SDW, Muse SV (2005). HyPhy: hypothesis testing using phylogenies. Bioinformatics.

[106] Darriba D, Taboada GL, Doallo R, Posada D (2012). jModelTest 2: more models, new heuristics and high-performance computing. Nat Methods.

[107] Guindon S, Gascuel O, Rannala B (2003). A simple, fast, and accurate algorithm to estimate large phylogenies by maximum likelihood. Syst Biol.

[108] Smith SA, Moore MJ, Brown JW, Yang Y (2015). Analysis of phylogenomic datasets reveals conflict, concordance, and gene duplications with examples from animals and plants. BMC Evol Biol.

[109] Zhang C, Rabiee M, Sayyari E, Mirarab S (2018). ASTRAL-III: polynomial time species tree reconstruction from partially resolved gene trees. BMC Bioinformatics.

[110] Huson DH, Bryant D (2006). Application of phylogenetic networks in evolutionary studies. Mol Biol Evol.

