# Supplementary material for: Identification and assessment of variable single-copy orthologous (SCO) nuclear loci for low-level phylogenomics: a case study in the genus Rosa (Rosaceae)
Source: BMC Evol Biol. 2019 Jul 24;19:152. doi: 10.1186/s12862-019-1479-z (PMC6657147; doi:10.1186/s12862-019-1479-z)
Supplement: Supplementary file 1 — : Supplementary figures. PDF file presenting the six supplementary figures cited in the core article. Each supplementary figure appears on a separate page and has its own caption. (PDF 2363 kb) [file 12862_2019_1479_MOESM1_ESM.pdf]

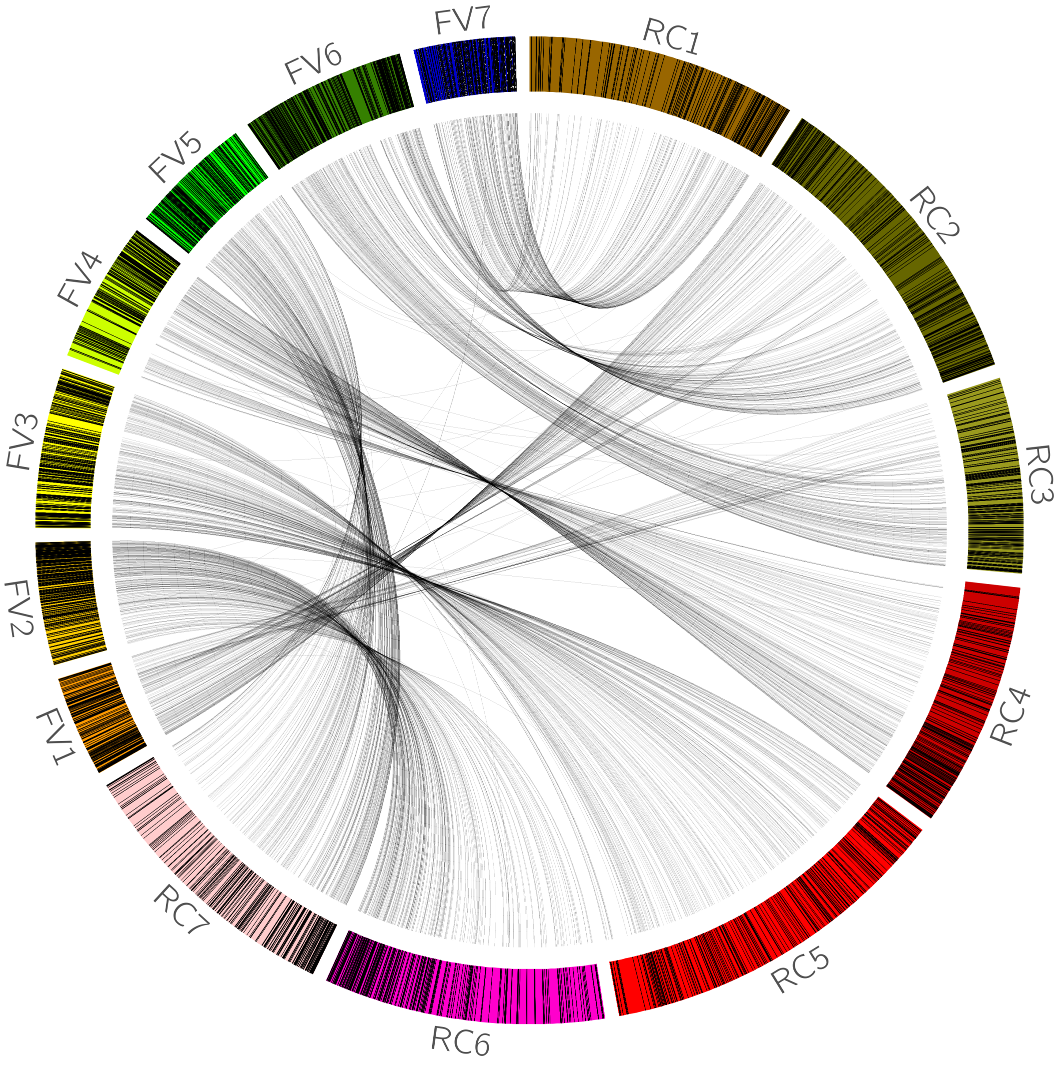

Supplementary Figure S1. Synteny analysis of the 1,784 Single-Copy Orthologs between the genome assemblies of *Fragaria vesca* and *R*. 'Old Blush'

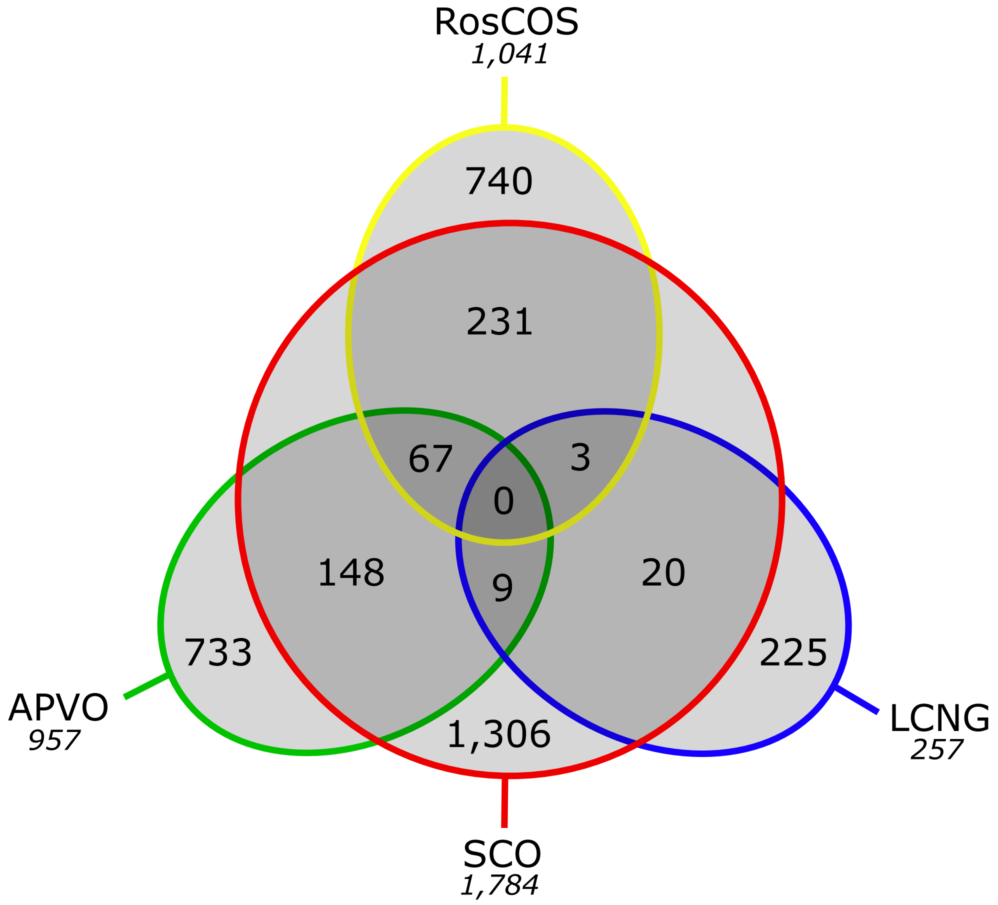

Supplementary Figure S2. Venn diagram showing the overlapping of the 1,784 Single-Copy Ortholgs (SCO) with 3 already published datasets. APVO refers to the *Arabidopsis*, *Populus*, *Vitis*, *Oryza* dataset [23], LCNG refers to the Low-Copy Nuclear Genes found by [27] and RosCOS corresponds to the Rosaceae Conserved Ortholog Set of markers [26].

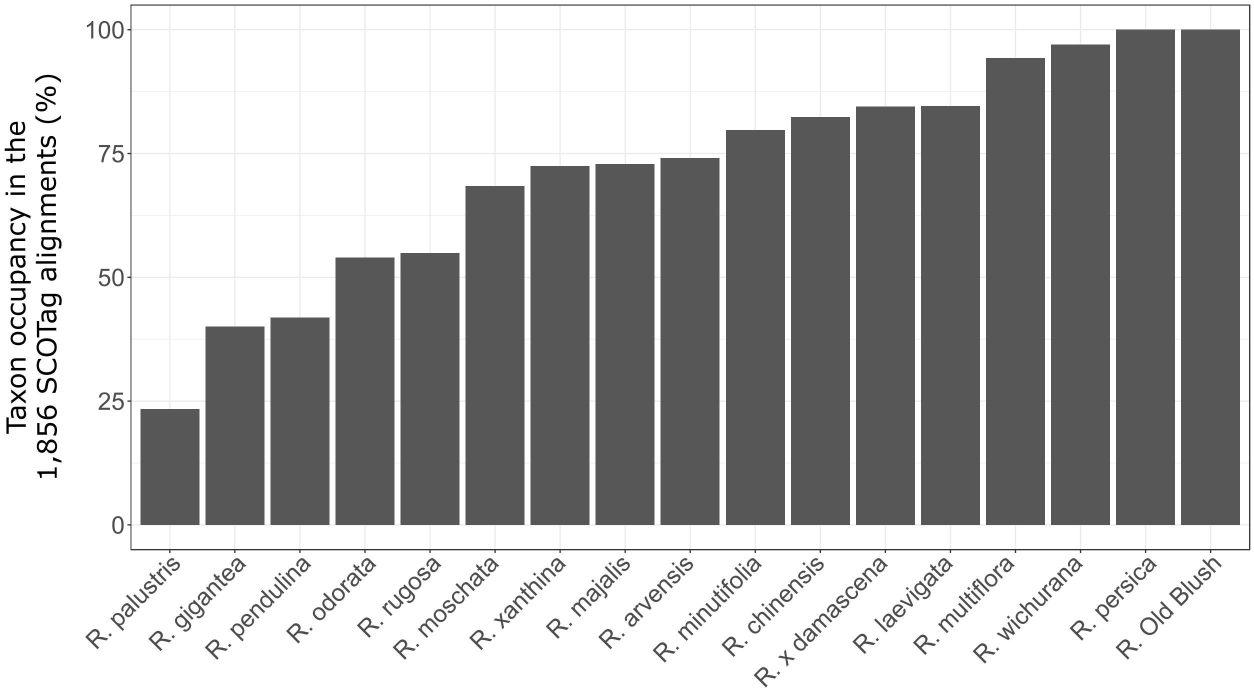

Supplementary Figure S3. Taxon occupancy of the *Rosa* ingroup for the 1,856 SCO_{Tag}s.

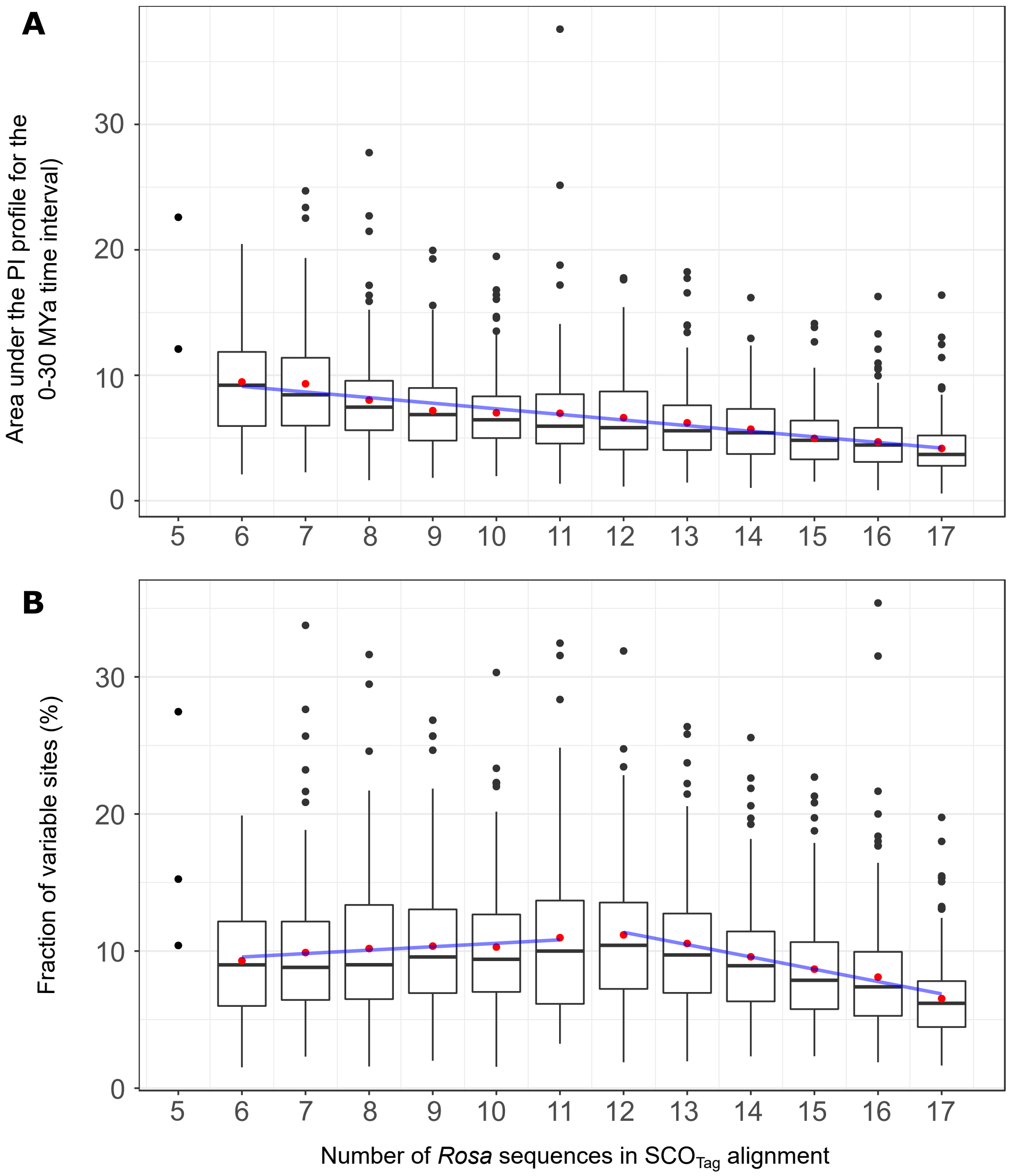

Supplementary Figure S4. Impact of taxon occupancy on A) the area under PI profiles for the 0-30 MYa time interval ([6-17]: y=11.8-0.45x, R^2=0.18) and B) the fraction of variable sites in SCO_{Tag} alignment ([6-11]: y=8.1 +0.25x, R^2=0.004; [12-17]: y=22.1-0.90x, R^2=0.11). Red dots indicate the mean values. Situations with less than 30 points were ploted but not used in the calculation of correlations.

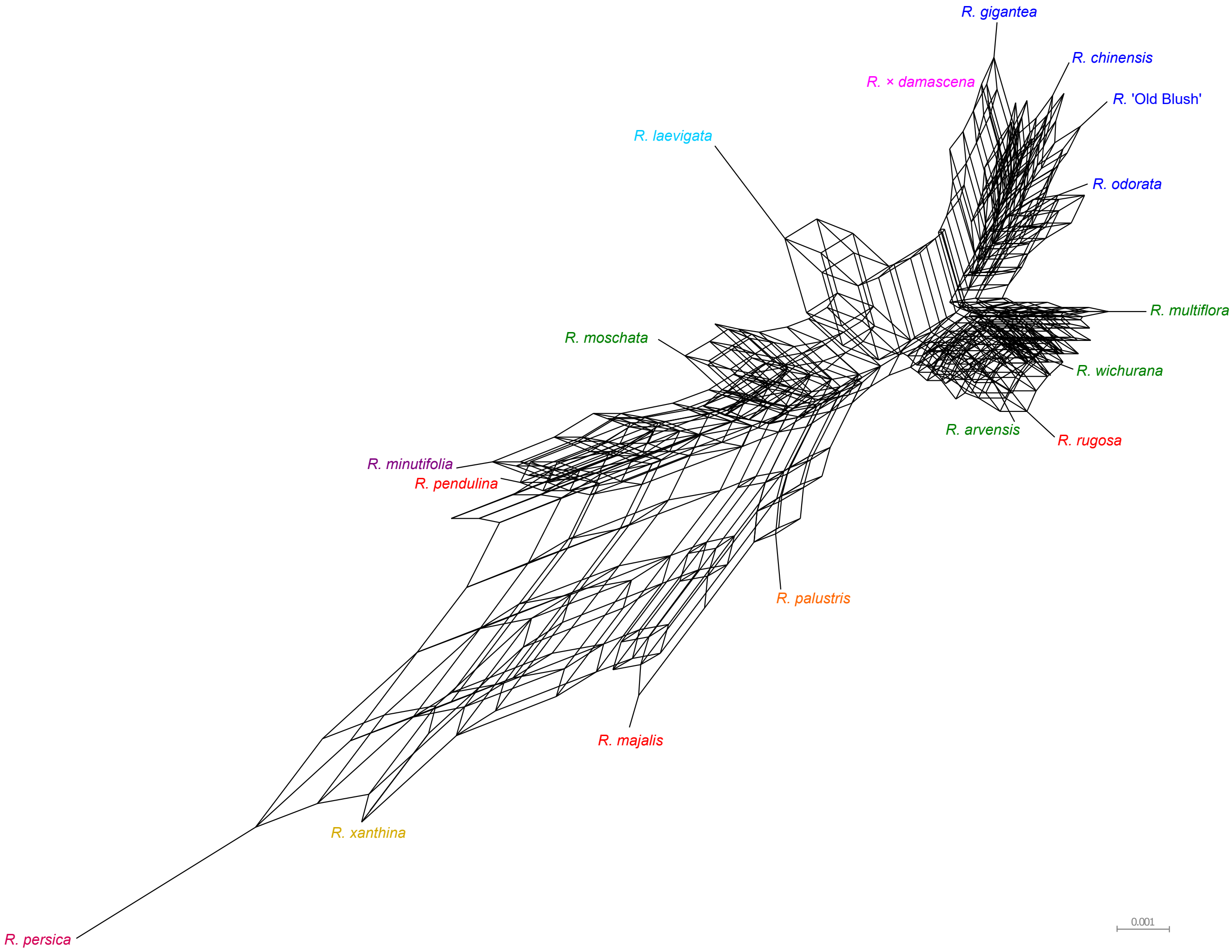

Supplementary Figure S6. Network representing the conflict between the 1,856 SCOTag trees for the *Rosa* ingroup. Species colors follow Figure 3.

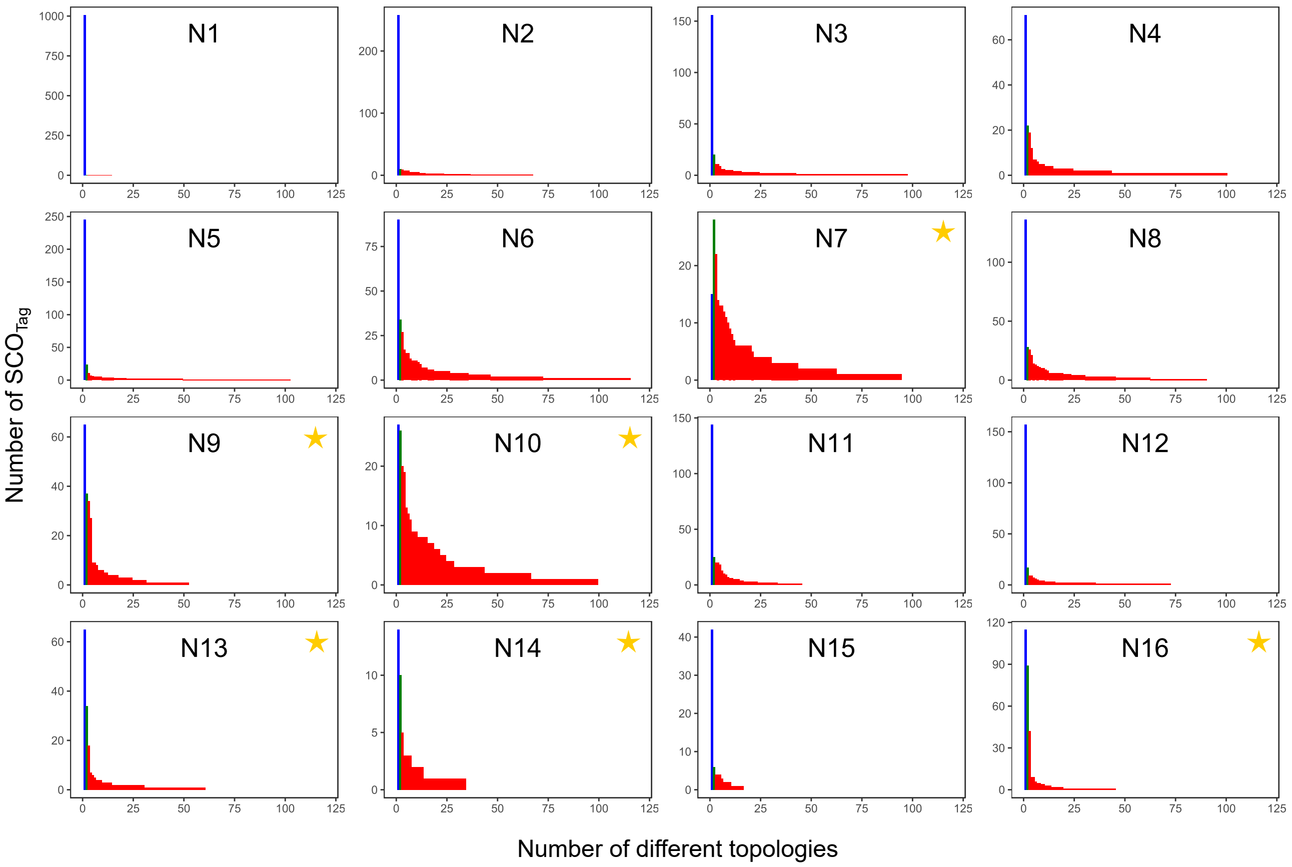

Supplementary Figure S5. Number of SCO$_{Tag}$s supporting each bipartition. Each plot title indicates the node name as shown in Figure 4. Blue represents the SCO$_{Tag}$s that support the species-tree bipartition (ie. concordant SCO$_{Tag}$s), green indicates the SCO$_{Tag}$s that agree with the main alternative bipartition (ie. main conflicting SCO$_{Tag}$s) and red corresponds to SCO$_{Tag}$s that support other alternative bipartitions (ie. other conflicting SCO$_{Tag}$s). Stars indicate nodes with significant conflicts, ie the proportion of SCO$_{Tag}$s supporting the main alternative bipartition is greater than 50% of the proportion of SCO$_{Tag}$s supporting the species-tree bipartition.
