# Supplementary material for: Identification and assessment of variable single-copy orthologous (SCO) nuclear loci for low-level phylogenomics: a case study in the genus Rosa (Rosaceae)
Source: BMC Evol Biol. 2019 Jul 24;19:152. doi: 10.1186/s12862-019-1479-z (PMC6657147; doi:10.1186/s12862-019-1479-z)
Supplement: Supplementary file 5 — : Word file containing the supplementary Table S1. (DOCX 14 kb) [file 12862_2019_1479_MOESM5_ESM.docx]

Table S1 Origin of plastid sequences used for phylogenetic inferences.

Plastid sequences were either obtained by target-assembly/Blast or retrieved from GenBank. Dashes indicate missing data.

| Species | *psbA-trnH* | *trnG* | *trnL* |
| --- | --- | --- | --- |
| *R. arvensis* | Recovered | Recovered | Recovered |
| *R. chinensis* | Recovered | Recovered | Recovered |
| *R. gigantea* | Recovered | Recovered | Recovered |
| *R. laevigata* | Recovered | Recovered | Recovered |
| *R. majalis* | Recovered | Recovered | Recovered |
| *R. minutifolia* | DQ778786 | Recovered | Recovered |
| *R. moschata* | Recovered | Recovered | Recovered |
| *R. multiflora* | Recovered | KJ575281 | KJ575162 |
| *R.* 'Old Blush' | Recovered | Recovered | Recovered |
| *R. odorata* | Recovered | Recovered | Recovered |
| *R. palustris* | DQ778798 | KJ575290 | DQ778877 |
| *R. pendulina* | Recovered | Recovered | Recovered |
| *R. persica* | Recovered | Recovered | Recovered |
| *R. rugosa* | Recovered | Recovered | Recovered |
| *R. wichurana* | Recovered | - | Recovered |
| *R.*× *damascena* | LC374596 | - | KT359474 |
| *R. xanthina* | Recovered | Recovered | Recovered |
| *F. vesca* | FJ493305 | FJ422324 | AF163559 |
